# Whole-genome and time-course dual RNA-Seq analyses reveal chronic pathogenicity-related gene dynamics in the ginseng rusty root rot pathogen *Ilyonectria robusta*

**DOI:** 10.1038/s41598-020-58342-7

**Published:** 2020-01-31

**Authors:** Yiming Guan, Meili Chen, Yingying Ma, Zhenglin Du, Na Yuan, Yu Li, Jingfa Xiao, Yayu Zhang

**Affiliations:** 1 Institute of Special Wild Economic Animal and Plant Science, Chinese Academy of Agricultural Sciences, Changchun, China; 2 Engineering Research Center of Chinese Ministry of Education for Edible and Medicinal Fungi, Jilin Agricultural University, Changchun, China; 3 National Genomics Data Center, Beijing Institute of Genomics, Chinese Academy of Sciences, Beijing, China; 4 BIG Data Center, Beijing Institute of Genomics, Chinese Academy of Sciences, Beijing, China; 5 CAS Key Laboratory of Genome Sciences and Information, Beijing Institute of Genomics, Chinese Academy of Sciences, Beijing, China; 6 University of Chinese Academy of Sciences, Beijing, China

**Keywords:** Fungal genomics, Effectors in plant pathology

## Abstract

*Ilyonectria robusta* causes rusty root rot, the most devastating chronic disease of ginseng. Here, we for the first time report the high-quality genome of the *I*. *robusta* strain CD-56. Time-course (36 h, 72 h, and 144 h) dual RNA-Seq analysis of the infection process was performed, and many genes, including candidate effectors, were found to be associated with the progression and success of infection. The gene expression profile of CD-56 showed a trend of initial inhibition and then gradually returned to a profile similar to that of the control. Analyses of the gene expression patterns and functions of pathogenicity-related genes, especially candidate effector genes, indicated that the stress response changed to an adaptive response during the infection process. For ginseng, gene expression patterns were highly related to physiological conditions. Specifically, the results showed that ginseng defenses were activated by CD-56 infection and persisted for at least 144 h thereafter but that the mechanisms invoked were not effective in preventing CD-56 growth. Moreover, CD-56 did not appear to fully suppress plant defenses, even in late stages after infection. Our results provide new insight into the chronic pathogenesis of CD-56 and the comprehensive and complex inducible defense responses of ginseng root to *I*. *robusta* infection.

## Introduction

Rusty root rot disease, caused by the *Ilyonectria destructans/Cylindrocarpon destructans* species complex, is the most devastating chronic disease of ginseng (*Panax ginseng* C. A. Meyer) and occurs in all ginseng growing areas^1^. With a cultivation history of more than 4,000 years, ginseng is the most valuable plant in traditional Chinese medicine. Currently, the major cultivation areas of ginseng are in China and South Korea; it is also grown in Japan, North Korea, Canada, and the United States. The northeastern region is the most important ginseng production area in China. Furthermore, the phylogeny and population genetics of ginseng suggest that Fusong town in Northeast China might be the domestication center of Asian ginseng, as the variety cultivated there is closest to wild ginseng^2^.

Ginseng rusty root rot is a soil fungal disease that can devastate ginseng crops. The pathogen exists not only in the soil and rhizosphere but also as an endophyte in the ginseng root^3^. Despite variation in the pathogenicity of members of the *C. destructans* species complex, *I. robusta* is the dominant pathogen with the highest proportion of ginseng rusty root pathogenicity (unpublished data), and the fungus is one of the main factors contributing to the difficulty in Asian ginseng continuous cropping. For example, the detection rate of rusty root rot in continuously cropped 2-year-old ginseng was reported to be 95.8%^4^. *I*. *robusta*, isolated from *Panax quinquefolium*, was first reported and described by Hildebr^5^ as *Ramularia robusta*. After several revisions, *Ramularia* changed from *Cylindrocarpon* to *Ilyonectria*^3,6–9^. Currently, the isolates of *Ilyonectria* species that infect ginseng are divided into four species: *I*. *robusta*, *I*. *mors-panacis*, *I*. *panacis*, and *I*. *crassa*^7^.

All parts of the ginseng root can be infected, with the infected root exhibiting reddish brown dry rot with deep gullies across the majority of the organ. However, infection stops with increased temperature; some parts of the ginseng root may recover, but the root retains the gullies and dry rot scars. Infected ginseng roots cannot be processed for sale because it is unclear how any fungal toxins present would be metabolized in humans. The *I*. *destructans* species complex can also infect some perennial herbs, such as *Panax quinquefolius* and *Narcissus*, and woody plants, such as *Populus*, *Quercus*, *Tilia* and *Prunus*^7,10^. However, the pathogen is not considered aggressive and may be an opportunistic plant root pathogen^6^. Previous studies on *I*. *destructans* have focused on systematics, molecular detection, and pathogenicity differentiation^6,11–14^.

The CD-56 strain used in this study is the first strain with a sequenced genome. It has been reported that the contents of iron and phenolic compounds are much higher in diseased tissue than in healthy tissue, which is believed to explain the rusty symptoms^15^. Additionally, Farh^16^ speculated that these symptoms develop as a result of the interaction between the pathogen and a non-resistant host. Although the genome and pathogenic mechanisms of this *Ilyonectria* have not been studied in detail, Gao Yuan^17^ applied transcriptome analyses to identify some resistance genes of *P*. *ginseng* induced by *I*. *destructans* in a time-course study.

The dual RNA-Seq approach has been widely used to explore the molecular mechanisms of plant–pathogen interactions. Kawahara^18^ reported both rice and fungal genes to be expressed at a relatively early stage of infection (24 h), with large amounts of rice and fungal transcripts produced during infection. Analyses of the cacao and *Moniliophthora perniciosa* transcriptomes during their unique biotrophic interaction showed that infection with *M*. *perniciosa* triggers massive metabolic reprogramming in diseased tissues^19^. In addition, mixed transcriptome analysis revealed significant differences in the transcriptional responses of banana to two different strains of pathogen (Foc1 and Foc4) at 48 h after inoculation^20^. Another study identified an array of factors and various strategies to facilitate host colonization and mitigate host defenses during *Brassica napus* infection with *Sclerotinia sclerotiorum*^21^. Lysøe^22^ and Boddu^23^ examined head blight disease, the most destructive disease of wheat and barley, through global gene expression of *Fusarium graminearum* from 24 h to 196 h postinoculation. Transcriptome profiling of *Fusarium solani* f. sp. *eumartii*, which causes dry rot of potato, and infected potato tubers also provided evidence of an inducible defense response at 24 h after pathogen infection^24^, and for late blight of potato, faster and stronger activation of defense-related genes was found to correlate with tuber resistance to *Phytophthora infestans*^25^.

There are various mechanisms of pathogen virulence and a wide range of plant immune responses. Several studies have reported that defense-related transcription factors, such as WRKY and YMB, and factors related to reactive oxygen species (ROS), the auxin pathway, and the ethylene pathway are manifestations of host plant resistance^26–28^. To recognize pathogens, hosts have evolved the receptor protein immunity system that identifies effectors and their associated molecular patterns (PAMPs/MAMPs), and infected plants can then initiate first-line innate PAMP-triggered immunity or second-line effector-triggered immunity^29–32^.

Here, we report the high-quality whole-genome sequence, candidate effectors and ginseng–pathogen interaction transcriptome analysis of *I*. *robusta* CD-56. The results provide a foundation for elucidating the mechanisms of the chronic pathogenicity of this pathogen. CD-56 initiates pathogenicity- and virulence-associated signaling pathways and possesses pathogenic mechanisms to suppress host defenses after three infection stages. Ginseng defends itself against CD-56 via expression of defense-related transcription factors and factors related to ROS, auxin, and ethylene pathways.

## Results

### Structure and general features of the *I*. *robusta* CD-56 genome

#### General features of the *I*. *robusta* CD-56 genome

We used PacBio sequencing technology to generate long reads to assemble the genome and high-quality Illumina short reads to correct assembly errors. The draft genome of CD-56 contains 53 contigs, with a total size of 58,742,480 bp and a GC content of 49.32%. In total, 17,438 coding sequences (CDSs) were predicted, of which 16,765 were validated by homology searches. The draft genome contains 221 tRNA genes, 57 rRNA genes, and 36 ncRNA genes. There are 8,699 CDSs (49.89%) that were assigned to one or more Gene Ontology (GO) functional classes (Fig. S1A). In total, 8,402 CDSs (48.18%) were assigned a function in Kyoto Encyclopedia of Genes and Genomes (KEGG) based on KEGG GENE alignment. Table 1 summarizes the general features of the draft genome.

**Table 1:** General features of the CD-56 genome assembly.

	CD-56
Genome assembly size	58,742,480 bp
Contig No.	53
Contig N50	3,405,880 bp
Contig N90	1,262,619 bp
GC content	49.32%
Gene No.	17,752
tRNA gene No.	221
rRNA gene No.	57
ncRNA gene No.	36
Total number of CDSs	17,438
Pseudo genes	29
Hypothetical proteins	8,730
CDSs with assigned function	11,250
CDSs assigned to KEGG pathways	8,402
CDSs assigned to GO function	8,699

According to KEGG pathway functional annotation analysis, ABC-transporter genes can be classified into ABCB, ABCC, ABCD and ABCG subfamilies, and genes involved in the two-component system are primarily related to osmolyte synthesis in response to hyperosmotic stress. The core elements of the mitogen-activated protein kinase (MAPK) signaling pathways are required for virulence in a wide array of fungal pathogens^33^. In CD-56, we predicted 25 homologs (62 genes in total) in the MAPK pathway and 11 homologs (22 genes in total) in the calcium pathway. Some common elements in the MAPK pathway that have been found in fungi pathogenic toward plants and human^34^ were identified in CD-56, as follows: (1) seven homologs of *ERK1*/2 (previously named *Fus3* and *Kss1*) in the MAPK pathway: *GNG* (previously named *Ste18*, *AUP68_13344*), *Cdc24* (*AUP68_02103*, *AUP68_17153*), *Cdc42* (*AUP68_01350*), *PAK1* (previously named *Ste20*, *AUP68_11641*, *AUP68_11642*), *Ste11* (*AUP68_01307*), *MAPK1_3* (previously named *Fus3/Kss1*, *AUP68_03166*, *AUP68_07501*, *AUP68_16124*), and *Bni1* (*AUP68_14625*); (2) 11 homologs of *p38* (previously named *Hog1*) in the MAPK pathway: *Ypd1* (*AUP68_03680*), *Cdc42* (*AUP68_01350*), *PAK1* (previously named *Ste20*, *AUP68_11641*, *AUP68_11642*), *Ssk1* (*AUP68_07001*), *Ste11* (*AUP68_01307*), *Ssk2/22* (*AUP68_05583*, *AUP68_08703*, *AUP68_02954*, *AUP68_04941*, *AUP68_14443*, *AUP68_04354*, *AUP68_09493*, *AUP68_02668*, *AUP68_12812*, *AUP68_04711*, *AUP68_14123*, *AUP68_13940*, *AUP68_04446*, *AUP68_07705*, *AUP68_07428*, *AUP68_05569*, *AUP68_13955*, *AUP68_04140*, *AUP68_07955*, *AUP68_07954*, *AUP68_00753*, *AUP68_15796*), *Pbs2* (*AUP68_06808*), *p38* (previously named Hog1, *AUP68_04006*, *AUP68_06362*), *Sko1* (*AUP68_09790*), *Msn2/4* (*AUP68_15493*), and *Mcm1* (*AUP68_06824*); and (3) five homologs of Mpk1 in the MAPK pathway: *Rh1* (*AUP68_16522*), *Pkc1* (*AUP68_02677*, *AUP68_12952*), *Bck1* (*AUP68_09749*, *AUP68_16215*), *Mkk1/2* (*AUP68_13277*, *AUP68_12069*), and *Fks2* (*AUP68_05335*, *AUP68_14756*, *AUP68_16064*). The majority of the core elements of *ERK1*/2 and *p38* in the MAPK pathway were identified in the CD-56 genome, especially the *p38* MAPK pathway. The pheromone/receptor pair genes *Ste2* (*AUP68_07997*) and *Ste3* (*AUP68_13310*) were also identified in CD-56, but genes encoding *HD1*/*HD2* transcription factors were not detected.

#### General features of CD-56 secretome protein genes

**Figure 1:**
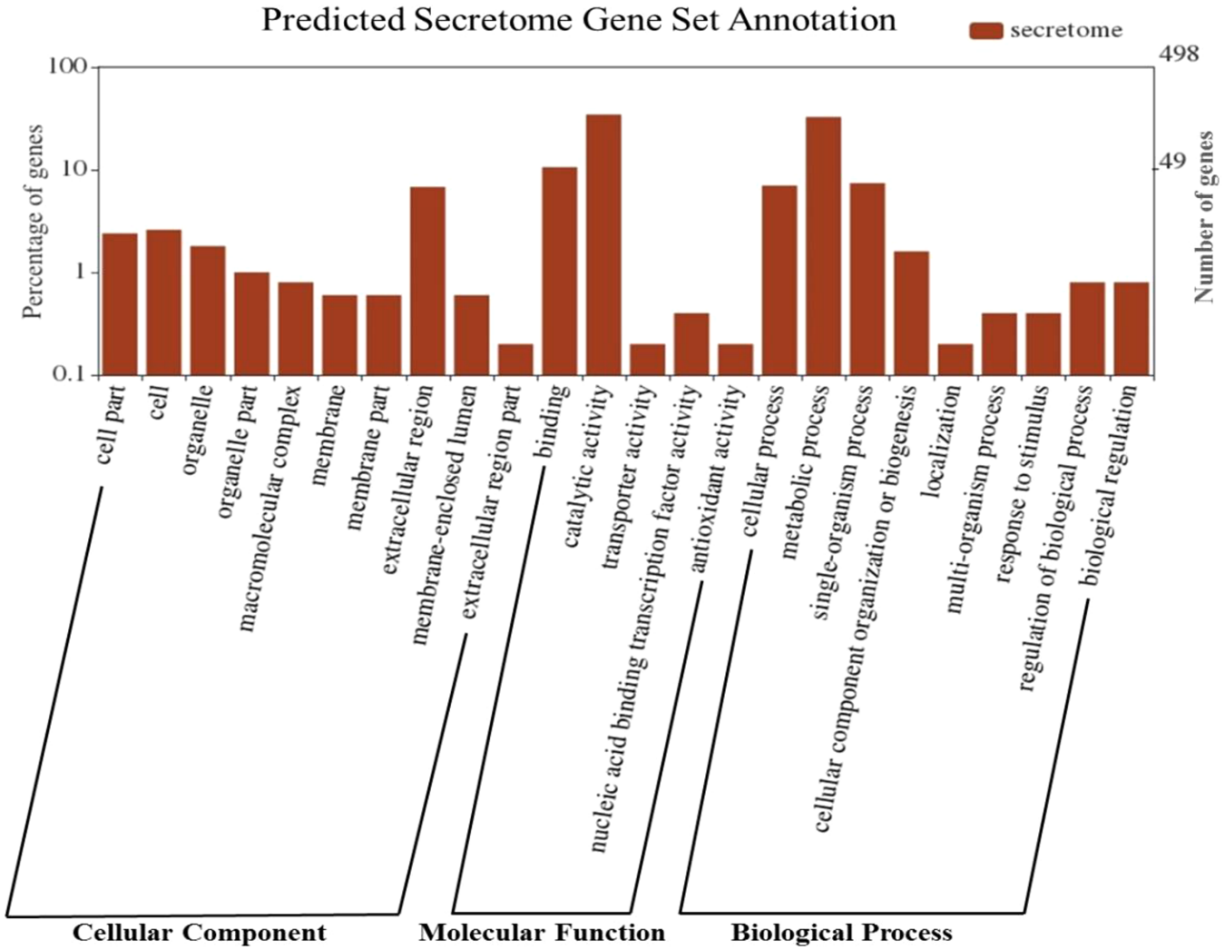
GO functional annotation analysis of the CD-56 predicted secretome gene set.

A subset of secretome protein genes from pathogens may determine the progression and success of infection^35^. We predicted 498 genes encoding secretome proteins in the CD-56 strain. A total of 451 genes were validated by homology search (nr database), and 207 genes were hypothetical genes. Genes with hits in the nr database are mainly from *Neonectria ditissima*, followed by *Nectria haematococca*, *Fusarium oxysporum* and several others; *Neonectria* and *Fusarium* are closely related to *Ilyonectria*. GO functional annotation analysis shows that most genes encode proteins related to extracellular region, cell part, cell and organelle functions at the cellular component level. Additionally, genes at the molecular function level are primarily related to catalytic activity, binding, nucleic acid binding transcription factor activity functions, and those at the biological process level are mainly involved in metabolic processes, cellular processes, single-organism process, biological regulation and cellular component organization or biogenesis (Fig. 1).

Among the predicted secretome proteins, some may have an important role in pathogenesis. In total, 121 secretome protein genes in CD-56 were assigned in the Pathogen–Host Interaction (PHI) database. The predicted pathogenicity-associated genes from CD-56 were classified into six phenotype-associated genes on the basis of their ability to modulate host responses and trigger plant cell death (based on the “Definition of phenotypes” in the PHI database^36^), as follows: (1) reduced virulence: disruption of 40 genes (34.40%) in the CD-56 genome could reduce virulence; (2) loss of pathogenicity: three genes (2.40%) in the CD-56 genome are predicted to be pathogenicity factors, though disruption of these genes would fail to cause disease; (3) effector: seven genes (5.60%) in the CD-56 genome are plant avirulence determinant genes, which are required for direct or indirect recognition of a pathogen only in resistant host genotypes possessing the corresponding disease-resistance gene; (4) unaffected pathogenicity: disruption of 55 genes (44%) in the CD-56 genome could reduce or result in no virulence; (5) increased virulence: disruption of three genes (2.40%) in the CD-56 genome could increase virulence; (6) mixed outcome: 13 genes (11.20%) in the CD-56 genome with uncharacterized functions might have one or more of the function types mentioned above. Details are presented in Table S1. In addition, proteins that are both high in cysteine content (≥6) and multiple tandem repeats (≥14) are predicted to be potential pathogenic effector proteins^37^, and 10 novel pathogenic effector candidate genes (*AUP68_00306*, *AUP68_01779*, *AUP68_02663*, *AUP68_05479*, *AUP68_07712*, *AUP68_11521*, *AUP68_13033*, *AUP68_13384*, *AUP68_14284*, and *AUP68_16048*) were identified in CD-56. These genes might be related directly to the infection of ginseng roots or indirectly to pathogenicity.

#### General features of CD-56 carbohydrate-active enzyme genes

The repertoire of carbohydrate-active enzymes (CAZymes) in fungal genomes is considered to have a strong relationship with the saprophytic lifestyle of these organisms^33^. The genome of the CD-56 strain is predicted to encode 129 CAZymes among the predicted secretome proteins, including the following: (1) 18 genes of the carbohydrate-binding module (CBM) family, which are from 10 subfamilies; (2) 17 genes of the carbohydrate esterase (CE) family, which are from six subfamilies; (3) 59 genes of the glucoside hydrolase (GH) family, which are from 32 subfamilies; (4) one gene from the glycosyl transferase (GT) family, which is from one subfamily; (5) 15 genes from the polysaccharide lyase (PL) family, which are from four subfamilies; and (6) 19 genes from the auxiliary activity (AA) family, which are from seven subfamilies. Details are shown in Table S2.

### Ginseng transcriptome reconstruction

#### Transcript assembly of the ginseng transcriptome

A total of 224,617 transcripts with lengths >= 200 bp were generated using Trinity software (Table S3) to assemble the pure ginseng transcriptome (sample IDs, Ginseng_1 and Ginseng_2). The mean transcript size is 779 bp, with an N50 of 1,296 bp. A set of non-redundant unigenes was obtained by retaining the longest transcript in a cluster as the representative and assigning all transcripts to 104,708 unigenes. Among these unigenes, the mean length is 681 bp, with an N50 of 1,107 bp. A total of 36,878 (36.22%) unigenes are longer than 500 bp and 17,786 (16.99%) are longer than 1,000 bp (Fig. S2). The sequencing reads were mapped to non-redundant unigenes, with mapping rates ranging from 63.45% to 65.55% (Table S4).

#### Functional annotation of ginseng assembled transcripts

Unigene sequences were aligned using BLAST searches against the NCBI nr and KEGG GENES databases, with a cutoff e-value of <1e^−5^. Among all unigenes, 34,773 (33.21%) and 27,277 (26.05%) matched with sequences in the NCBI nr and KEGG GENES databases, respectively. The large percentage of unigenes with unknown function was expected because of the paucity of sequences from phylogenetically closely related species in public databases.

Functional classification of the unigenes was conducted by GO analysis, and 19,791 (19,791/104,708) unigenes were successfully assigned to at least one GO term (Fig. S1B). The unigenes were then classified into three categories: biological process, cellular component, and molecular function. In the cellular component category, the major represented subcategories were cell (GO: 0005623) and cell part (GO: 0044464). In the biological process category, cellular process (GO: 0009987) was the most represented GO term, followed by metabolic process (GO: 0008152).

### Dual RNA-Seq analysis of *I*. *robusta*-infected ginseng cells

#### General features of dual RNA-Seq

**Figure 2:**
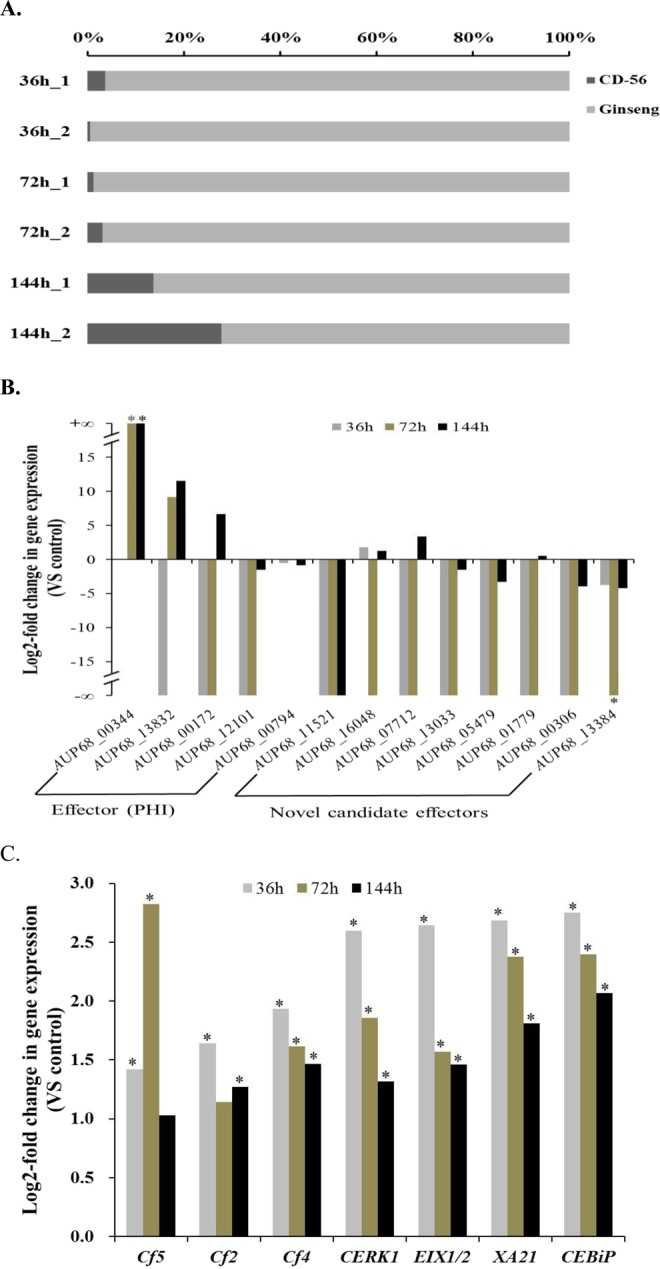
Dual RNA-Seq captures the expressed transcript repertoire of infected cells. (**A**) Representative mapping statistics from *I*. *destructan*s CD-56-infected ginseng root cells. (**B**) Infecting CD-56 cells at 36 hours (36 h), 72 hours (72 h) and 144 hours (144 h) activated some effector genes and inhibited other effector genes (+${\rm{\infty }}$ denotes halted effector gene expression under the control condition, −${\rm{\infty }}$ denotes halted effector gene expression after infection). (**C**) Invaded host cells at 36 hours (36 h), 72 hours (72 h) and 144 hours (144 h) activated ginseng cell-surface PRRs (pattern-recognition receptors; note that a transcript expression level is defined as the average of the transcript expression values of the two replicate samples; the gene expression level is defined as the sum of all transcript expression values, which were assigned to a same ko No.). Data in panels B and C represent log2 fold changes calculated by Cuffdiff for 2 biological replicates. The asterisk (‘*’) in panels B and C indicates that the gene/transcript expression value after infection was significantly different from the control condition.

We conducted dual RNA-Seq to reveal the signaling events in the host (ginseng) directly modulated by the pathogen (CD-56). To assess the reliability of RNA-Seq, each biological sample had two replicates. Reads from CD-56 cells or ginseng cells alone were mapped to the CD-56 assembled genome or the ginseng assembled transcript dataset. The levels of individual transcripts were highly consistent between the two biological replicates (Pearson’s correlation coefficient r ∈[0.8636, 0.9884], Fig. S3), except for the CD-56 sample at 72 h after inoculation (Pearson correlation coefficient r = 0.5461). We compared representative mappable reads to assess the coverage of the CD-56 and ginseng transcripts. Initially, the proportion of CD-56 reads ranged from 0.46% to 27.71%, which then increased rapidly to approximately 20% by 144 h after inoculation (Fig. 2A). This reflected the intracellular replication of the pathogen and confirmed the predicted excess of plant cell RNA over fungal cell RNA^38^. The observed changes in the proportions of reads were indicative of differentially expressed genes (DEGs) in both organisms during infection: (1) in CD-56 cells, the expression levels of some virulence-associated genes increased and some decreased after inoculation (Fig. 2B and Table S5); (2) in infected ginseng cells, expression of genes encoding cell-surface pattern-recognition receptors (PRRs) was activated after inoculation (Fig. 2C and Table S5).

**Figure 3:**
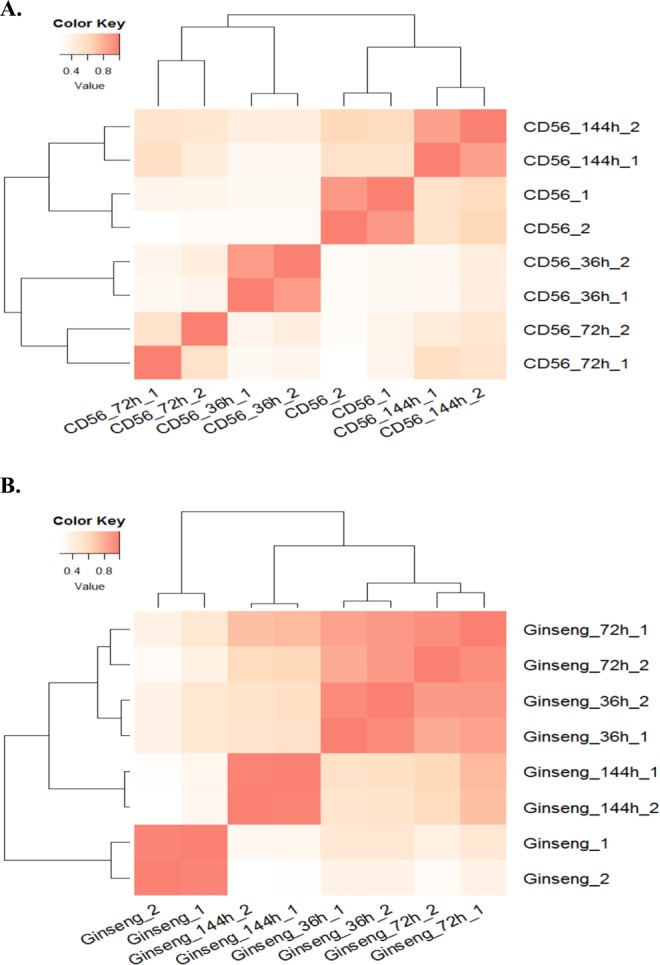
CD-56 and ginseng gene expression profile hierarchical cluster analyses based on Pearson correlation. Pearson correlation of gene expression profiles was used to define the similarity in gene expression profiles among 8 different samples. CD56_1 & CD56_2 and Ginseng_1 & Ginseng_2 are normal condition samples as the control group. (**A**) CD-56 gene expression profile hierarchical cluster analysis. (**B**) Ginseng gene expression profile hierarchical cluster analysis.

Analyses of the CD-56 gene expression profile indicated that many genes initially expressed were suppressed at 36 and 72 h after inoculation, and the number of genes with suppressed expression increased over time after infection. In ginseng, many genes that were initially expressed were also suppressed after infection, with the number of genes with suppressed expression initially increasing after infection and then decreasing over time. The CD-56 and ginseng transcriptome samples could be separated into two physiological groups: normal (control group, mock infected), and infected (three time points during infection, each with two replicate samples). Cluster analysis indicated similar gene expression patterns between the two replicates of each sample (Fig. 3). Additionally, the CD-56 gene expression patterns in the control and at 144 h after inoculation were similar (Fig. 3A). Unlike CD-56, ginseng showed gene expression patterns that were strongly related to physiological conditions (Fig. 3B). The three infection periods of CD-56 could be separated into two stages: 36 h and 72 h after inoculation as the initial infection stage and 144 h after inoculation as the adaptive infection stage. Cluster analysis indicated similar ginseng gene expression patterns between the 36 h and 72 h defense periods, consistent with the CD-56 gene expression patterns (Fig. 3). The three defense periods of ginseng could be separated into two stages: 36 and 72 h after inoculation represented the initial defense stage, and 144 h after inoculation represented the adaptive defense stage. The two defense stages of ginseng were similar to those of CD-56. Overall, the gene expression profile cluster analysis results showed that the infection response of CD-56 tended to return to the normal gene expression pattern by 144 h after inoculation and that ginseng showed a relatively consistent defense response (Fig. 3).

**Figure 4:**
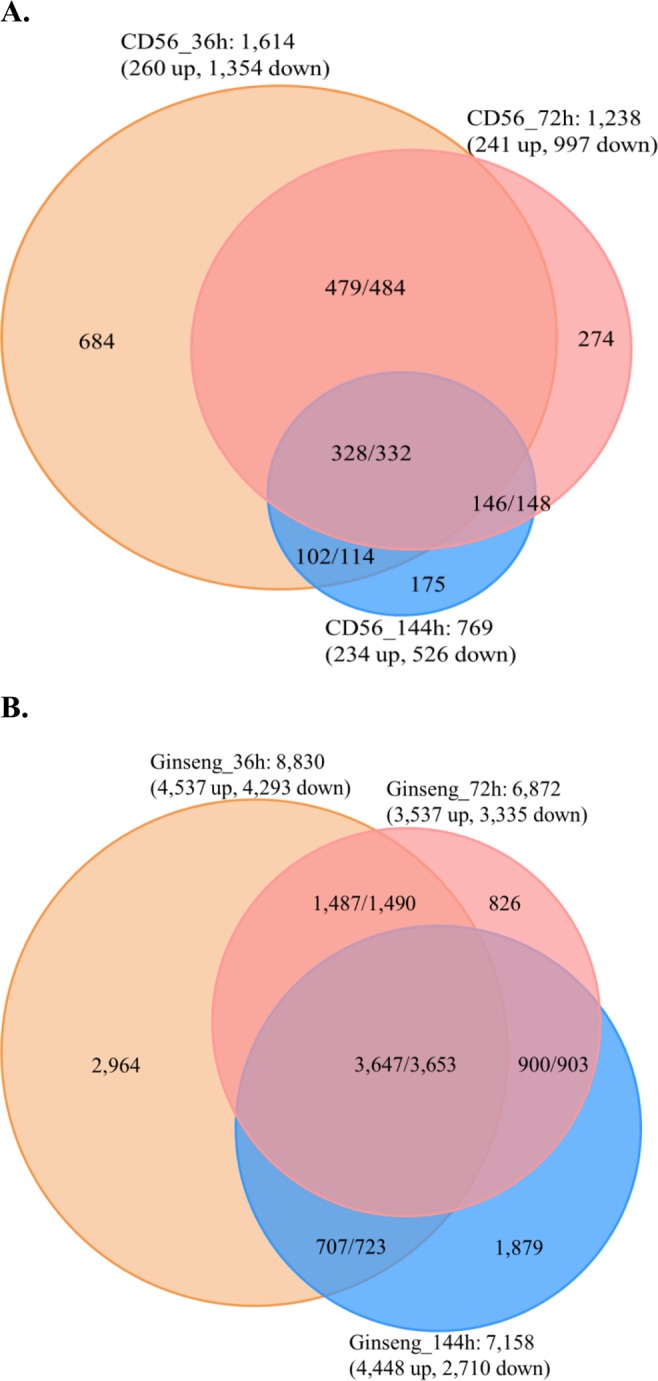
DEG numbers in the 3 infection periods for CD-56 and ginseng compared with the control. Note: (1) down indicates that a gene was significantly down-regulated compared to the control; (2) up indicates that a gene was significantly up-regulated compared to the control; (3) two numbers, formatted as A/B, for intersections indicate gene numbers with uniform regulation directions (**A**, for example, both up- and both down-regulated genes between 36 h and 72 h of infection) to total gene number (**B**).

We also used Cuffdiff^39^ and edgeR^40^ to identify genes exhibiting significant differences in expression levels between pairs of conditions, which allowed us to identify DEGs in CD-56 and ginseng at the three infection stages compared with their respective controls. In CD-56, there were 1,354 down-regulated genes and 260 up-regulated genes at 36 h after inoculation, 997 down-regulated genes and 241 up-regulated genes at 72 h after inoculation, and 526 down-regulated genes and 243 up-regulated genes at 144 h after inoculation (Fig. 4A). In ginseng, there were 4,537 down-regulated genes and 4,293 up-regulated genes at 36 h after inoculation, 3,537 down-regulated genes and 3,335 up-regulated genes at 72 h after inoculation, and 4,448 down-regulated genes and 2,710 up-regulated genes at 144 h after inoculation (Fig. 4B). Moreover, 684 genes (up to 42.38% of DEGs) in CD-56 cells were specifically activated at 36 h after inoculation, which was the highest proportion of activated genes during infection (Fig. 4A). A similar phenomenon was observed in the ginseng transcriptome (Fig. 4B). With regard to intersecting DEGs, the directions of regulation exhibited high uniformity (stably up- and down-regulated DEG proportions ranged from 95.90% to 99.84%, except an outlier at 89.47%) both in CD-56 and ginseng (Fig. 4). The transcriptome of CD-56 displayed significant regulation at the initial infection stage compared with the control but weaker regulation at the adaptive infection stage. The transcriptome of ginseng initially showed significant regulation upon infection compared with the control, but this regulation rapidly weakened in subsequent defense stage. However, some recovery of this transcriptome regulation was found at the final defense stage. In general, the significant changes during the initial infection/defense stages might be related to the stress response of ginseng. In CD-56, there was an initial down-regulation of genes, but the number of down-regulated genes decreased over time after infection, suggesting that inhibition of fungal cells gradually diminished with time. In ginseng, the number of up-regulated genes decreased and then increased with time after infection, though the number of down-regulated genes only decreased. This suggests a relatively long process of sustained defense in host ginseng cells.

Cluster analysis indicated similar DEG expression patterns between the two replicates of each sample (Fig. S4). DEG expression patterns were also similar between the 72 h and 144 h samples in CD-56, forming a cluster (Fig. S4A). Although DEG expression patterns were very similar between the two replicates of the 36 h CD-56 samples, they differed from those in other samples and formed a separate group (Fig. S4A). The results of this cluster analysis differed from those of the full gene expression profile cluster analysis (Fig. 3A). Indeed, the gene expression profile cluster analysis results suggested that the gene expression pattern of CD-56 tended to slowly return to a normal pattern (similar to that in the control) by 144 h after inoculation but that ginseng showed a relatively consistent defense response (Figs. 3A and S4A). Unlike CD-56, ginseng exhibited DEG expression patterns that were strongly related to its physiological condition (Fig. S4B), similar to the results of the full gene expression profile cluster analysis (Fig. 3B). In summary, the dual RNA-Seq analysis (Figs. 3 and S4) indicated that fungal CD-56 cells gradually recovered to normal conditions after infecting ginseng cells but that ginseng cells were continuously inhibited during the course of infection.

#### Pathogenicity of *I*. *robusta* CD-56 infection-related genes

GO annotation analysis of CD-56 genes related to infection. The GO annotation analysis of the CD-56 DEGs is shown in Fig. S5. Comparison of the enriched GO classes among the three infection stages indicated that the stage at 72 h after inoculation represented an intermediate stage. Some of the proteins encoded by DEGs at this stage overlapped with those encoded by DEGs at 36 h and 144 h after inoculation, though no overlap between 36 h and 144 h after inoculation was observed. The GO class distribution changed from transport- to cell aging-associated functions during the 144 h after inoculation. Regarding enriched GO function of DEGs, the proportion of down-regulated genes in CD-56 peaked at 72 h after inoculation (88.27%). From a cellular function perspective, CD-56 cells displayed a change in function at 72 h after inoculation.

Virulence-associated pathways in CD-56. KEGG pathway enrichment analysis showed that at 36 h after inoculation, DEGs were enriched in the ribosome pathway, pathogenic pathway, antigen processing and presentation pathway, alanine, aspartate, and glutamate pathways, nitrogen metabolism pathway, and Parkinson’s disease pathway. DEGs at the 72 h infection stage were enriched in disease-associated pathways, alanine, aspartate, and glutamate metabolism pathways, and the neuroactive ligand-receptor interaction pathway. At 144 h after inoculation, DEGs were enriched in amino sugar and nucleotide sugar metabolism pathways, fructose and mannose metabolism pathways, the pentose and glucuronate interconversion pathway, the nitrogen metabolism pathway, and the citrate cycle (TCA cycle) pathway. The KEGG pathway enrichment results are presented in Table 2.

**Table 2:** KEGG pathway enrichment analysis of CD-56 DEGs at three infection stages.

KEGG Pathway ID	Pathway name	M/N^a^	36 h VS control		72 h VS control		144 h VS control	
			m/n^b^	p-value	m/n^b^	p-value	m/n^b^	p-value
ko03010	Ribosome	119/4,400	39/463	2.10E-11^*^	8/353	0.75065	4/234	0.885899
ko05130	Pathogenic Escherichia coli infection	20/4,400	8/463	5.67E-04^*^	3/353	0.212972	1/234	0.665593
ko04612	Antigen processing and presentation	22/4,400	8/463	0.00119^*^	3/353	0.256954	1/234	0.700374
ko00250	Alanine, aspartate and glutamate metabolism	61/4,400	14/463	0.003575^*^	11/353	0.008216^*^	6/234	0.103376
ko00910	Nitrogen metabolism	56/4,400	13/463	0.004437^*^	10/353	0.012317	8/234	0.008793^*^
ko05012	Parkinson’s disease	47/4,400	11/463	0.008059^*^	10/353	0.003417^*^	6/234	0.036624
ko04930	Type II diabetes mellitus	15/4,400	2/463	0.478804	5/353	0.004943^*^	3/234	0.042036
ko05016	Huntington’s disease	68/4,400	12/463	0.04901	12/353	0.007021^*^	5/234	0.293932
ko05010	Alzheimer’s disease	60/4,400	10/463	0.093754	11/353	0.007239^*^	6/234	0.097288
ko04080	Neuroactive ligand-receptor interaction	6/4,400	2/463	0.124591	3/353	0.008526^*^	1/234	0.279695
ko00520	Amino sugar and nucleotide sugar metabolism	81/4,400	9/463	0.484646	9/353	0.198799	12/234	0.001033^*^
ko00051	Fructose and mannose metabolism	180/4,400	28/463	0.02083	23/353	0.016129	19/234	0.00292^*^
ko00040	Pentose and glucuronate interconversions	44/4,400	4/463	0.695087	5/353	0.275757	7/234	0.007794^*^
ko00020	Citrate cycle (TCA cycle)	45/4,400	6/463	0.334514	5/353	0.291727	7/234	0.008823^*^

^a^M, number of genes assigned to the corresponding KEGG pathway in the genome; N, number of genes assigned to at least one KEGG pathway in the genome.

^b^m, number of DEGs assigned to the corresponding KEGG pathway in the pairwise comparison; n, number of DEGs assigned to at least one KEGG pathway in the pairwise comparison.

^*^The asterisk indicates DEGs enriched significantly in a KEGG pathway corresponding to that infection stage.

After infection of the host, the enriched pathways in CD-56 changed from protein synthesis and pathogenicity-related pathways to disease-associated pathways and finally to the regulation of sugar and energy metabolism. At 36 h after inoculation, all DEGs encoding ribosome proteins were activated except for the gene encoding the L36e ribosomal protein (*AUP68_17121*); however, most DEGs recovered to normal levels at later stages of infection, and some gradually became suppressed. DEGs in the pathogenic pathway and antigen processing and presentation pathway were down-regulated at 36 h after inoculation, though some exhibited gradual increases in expression over time. This indicated that the expression levels of genes encoding ribosome components and pathogenicity-associated proteins in CD-56 gradually returned to normal levels (similar to those in the control) after infection. At 144 h after infection, the enrichment in sugar metabolism and energy metabolism was mainly associated with the synthesis of cell wall components. Chitin is one of the main components of the fungal cell wall, and its synthesis is catalyzed by *CHS1*^41,42^. Expression of *CHS1* was down-regulated at 36 h after inoculation, recovered to normal levels at 72 h, and was up-regulated at 144 h, indicating that chitin synthesis was initially inhibited in CD-56 but activated several days after the initial infection.

DEGs encoding proteins localized at the cytomembrane and responding to pheromone, cell wall stress, high osmolarity, and starvation signals were suppressed at 36 h after inoculation and slightly up-regulated at 72 h after inoculation, recovering to normal levels at 144 h after inoculation. The suppression phenomenon shifted from the cytomembrane to intracellular parts after infection. Interestingly, *Ste12* (*AUP68_08252*, *AUP68_13264*), which encodes a master regulator of invasive growth in plant pathogenic fungi to control fungal virulence downstream of the pathogenic MAPK cascade^43^, did not show significantly different regulation among the three infection stages. In addition, DEGs involved in the calcium signaling pathway, which indirectly affects proliferation, fertilization, learning, and memory, were also suppressed at 36 h after inoculation, recovered to normal levels at 72 h, but tended to be suppressed at 144 h.

**Figure 5:**
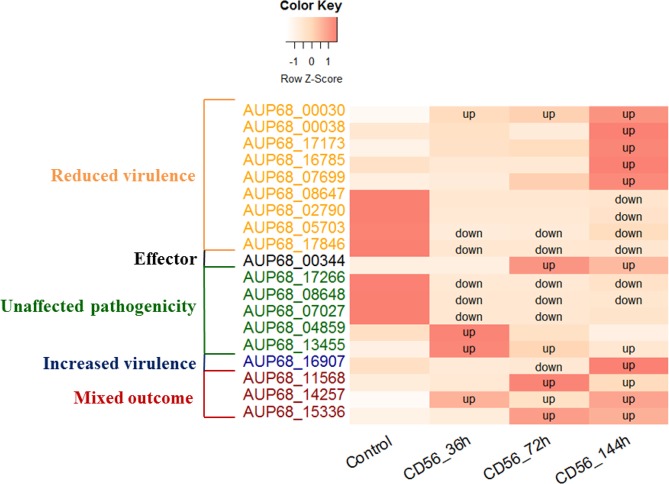
The dynamic change tendency of DEGs with hits in the PHI database compared to the control. Note that (1) down indicates that a gene was significantly down-regulated compared to the control; (2) up indicates that a gene was significantly up-regulated compared to the control.

*I*. *robusta* CD-56 candidate effectors. The majority of DEG secretome protein genes were down-regulated, and the proportion of down-regulated DEGs decreased gradually. However, the down-regulated DEGs that were identified in the PHI database were relatively stable in the three infection stages, whereas the number of up-regulated DEGs increased gradually (Table 3). Compared to the control, the predicted PHI effectors that were significantly regulated (Fig. 5) were as follows: (1) reduced virulence: *AUP68_00030*, which was up-regulated in all three infection stages, *AUP68_05703* and *AUP68_17846* were down-regulated in all three infection stages, especially *AUP68_17846*, which were not expressed in the 36 and 72 h infection periods, *AUP68_00038*, *AUP68_17173*, *AUP68_16785* and *AUP68_07699* were up-regulated at the 144 h infection period, and *AUP68_08647* and *AUP68_02790* were down-regulated in the 144 h infection period; (2) effector: *AUP68_00344* was not expressed at the initial infection stage, though it was expressed and up-regulated in the adaptive infection stage; (3) unaffected pathogenicity: *AUP68_17266*, *AUP68_08648*, and *AUP68_07027* were down-regulated and ceased in the 36 and 72 h infection periods, *AUP68_17266* and *AUP68_08648* remained down-regulated in the 144 h infection period, *AUP68_04859* and *AUP68_13455* were up-regulated in the initial infection stage, and *AUP68_13455* remained up-regulated at the adaptive infection stage; (4) increased virulence: *AUP68_16907* was down-regulated in the 72 h infection period but up-regulated in the 144 h infection period; (5) mixed outcome: *AUP68_14257* was up-regulated in all three infection periods, but *AUP68_11568* and *AUP68_15336* were up-regulated only in the adaptive infection stage. The novel predicted effector *AUP68_13384* was the only DEG with halted expression (significantly down-regulated) after 72 h of infection, which might indicate that it is not a ginseng-specific effector. The details are provided in Table 3.

**Table 3:** The distribution of CD-56 DEGs for diverse secrectome types.

	36 h VS control			72 h VS control			144 h VS control		
	up	down	total	up	down	total	up	down	total
Genome	260	1,354	1,614	241	997	1,238	243	526	769
Secretome	9	26	35	10	23	33	12	13	25
Effectors hit in PHI	4	5	9	6	6	12	11	6	17
CAZyme	4	2	6	7	2	9	12	2	14
Novel effectors	0	0	0	0	1	1	0	0	0

Regarding reduced-virulence DEGs, *AUP68_00030* was the predominantly expressed gene, and it was up-regulated in all three infection periods. The effector gene *AUP68_00344* was activated and up-regulated in the adaptive infection stage, indicating that it might be recognized by the corresponding ginseng disease-resistance gene, a host-specific toxin gene. For unaffected-pathogenicity genes, although the predominant regulatory mechanism appears to be down-regulation, expression levels were predominantly up-regulated. With regard to increased-virulence genes, *AUP68_16907* was down-regulated at 72 h after infection and up-regulated to a very high level at 144 h. Last, significantly regulated mixed-outcome genes were uniformly up-regulated. In general, genes with different types of genetic functions showed corresponding toxin peaks at different times.

**Figure 6:**
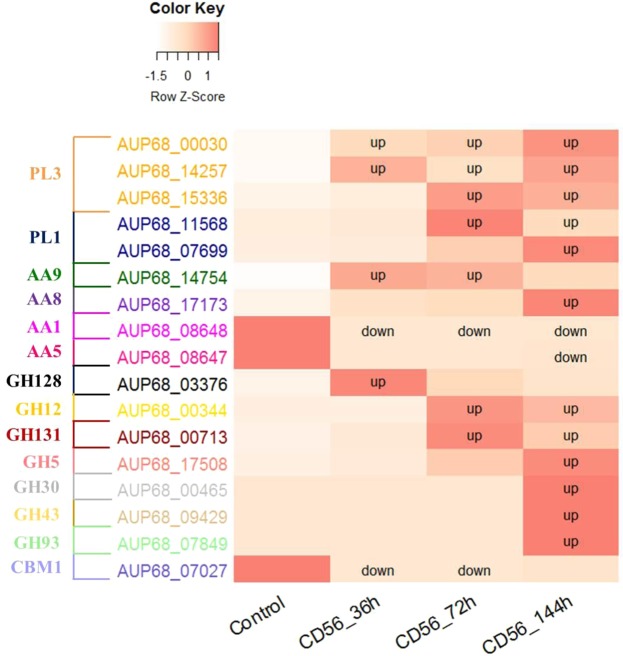
The dynamic change tendency of DEGs encoding CAZymes compared to the control. Note that (1) down indicates that a gene was significantly down-regulated compared to the control; (2) up indicates that a gene was significantly up-regulated compared to the control.

Pathogenicity genes among CAZymes. In total, 17 DEGs encoding CAZymes were identified in the three infection stages. Although expression of most of these genes was up-regulated, the AA1 subfamily gene *AUP68_08648* (switched off after 36 and 72 h of infection), the AA5 subfamily gene *AUP68_08647*, and the CBM1 subfamily gene *AUP68_07027* (switched off after 36 and 72 h of infection) exhibited down-regulated expression (Fig. 6). Moreover, lack of expression was observed for four and three genes at 36 and 72 h after infection, respectively. The following results were found for DEGs with up-regulated expression: (1) genes from PL3, AA9 and GH128 subfamilies at 36 h after infection; (2) genes from PL3, PL1, AA9, GH12, and GH131 subfamilies at 72 h after infection; and (3) genes from PL3, PL1, AA8, GH12, GH131, GH5, GH30, GH43 and GH93 subfamilies at 144 h after infection. Both the number and expression levels of genes indicated predominant up-regulation for CAZymes, with both increasing with infection time. The primarily associated CAZymes that might relate to the saprophytic lifestyle of CD-56 are PL and GH subfamily genes.

#### Expression of defense-related genes in ginseng

**Figure 7:**
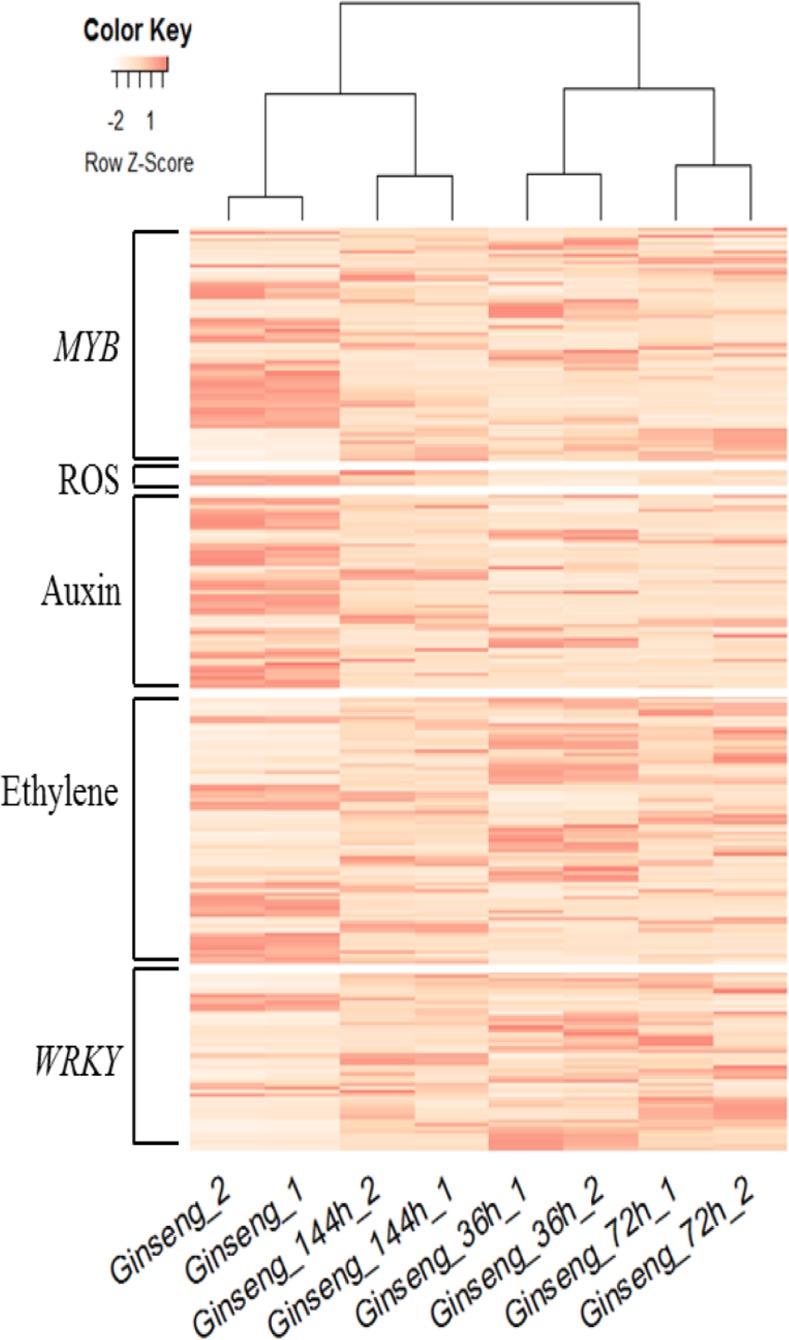
Ginseng defense-related DEGs (except plant–pathogen interaction pathway DEGs) expression profile hierarchical cluster analysis based on Pearson correlation. Pearson correlation of gene expression profiles was used to define the similarity in gene expression profiles among 8 different samples. Ginseng_1 and Ginseng_2 are normal condition samples as the control group.

To investigate the responses of defense-related genes in ginseng, we focused on six types of DEGs: those encoding components of the plant–pathogen interaction pathway (KEGG map04626), those related to ROS, auxin, and ethylene, and those encoding MYB and WRKY transcription factors^8,44–47^. In the plant–pathogen interaction pathway, cell-surface PRRs perceive pathogens and are key components of the primary response against invading pathogens^31^. Many genes encoding cell-surface PRRs were initially up-regulated in ginseng roots upon infection, but levels then gradually decreased (Fig. 2C). In the ethylene, WRKY, and plant–pathogen interaction pathways, there were more up-regulated genes than down-regulated genes at all three infection stages (Table 4), indicating that the ginseng root mounted a positive defensive response against the pathogen. In addition, there were more down-regulated than up-regulated auxin-related genes, indicating that the growth of ginseng was inhibited. For genes related to ROS and those encoding MYB transcription factors, there were more up-regulated than down-regulated genes at 72 h after inoculation, possibly because of a delayed response to the pathogen. Cluster analysis of the defense-related DEGs confirmed similar gene expression patterns between the two replicates of each sample and similar DEG expression patterns between control samples and samples at 144 h after inoculation (Fig. 7). These results indicate that DEGs during the defense response tended to return to normal expression levels by 144 h after inoculation, which was different from the trends observed across the entire transcriptome (Fig. 3B).

**Table 4:** Number of defense-related DEGs in various categories in ginseng.

	36 h VS control		72 h VS control		144 h VS control	
	up	down	up	down	up	down
Total DEGs	4,537	4,293	3,537	3,335	4,448	2,710
MYB	21	20	24	22	18	15
Reactive oxygen species	1	4	2	1	3	0
Auxin	6	34	3	31	4	23
Ethylene	35	20	30	16	31	13
WRKY	36	7	37	4	27	1
Plant–pathogen interaction	202	115	159	94	194	60

### Relative expression of secretome protein genes by quantitative real-time (qRT-PCR)

**Figure 8:**
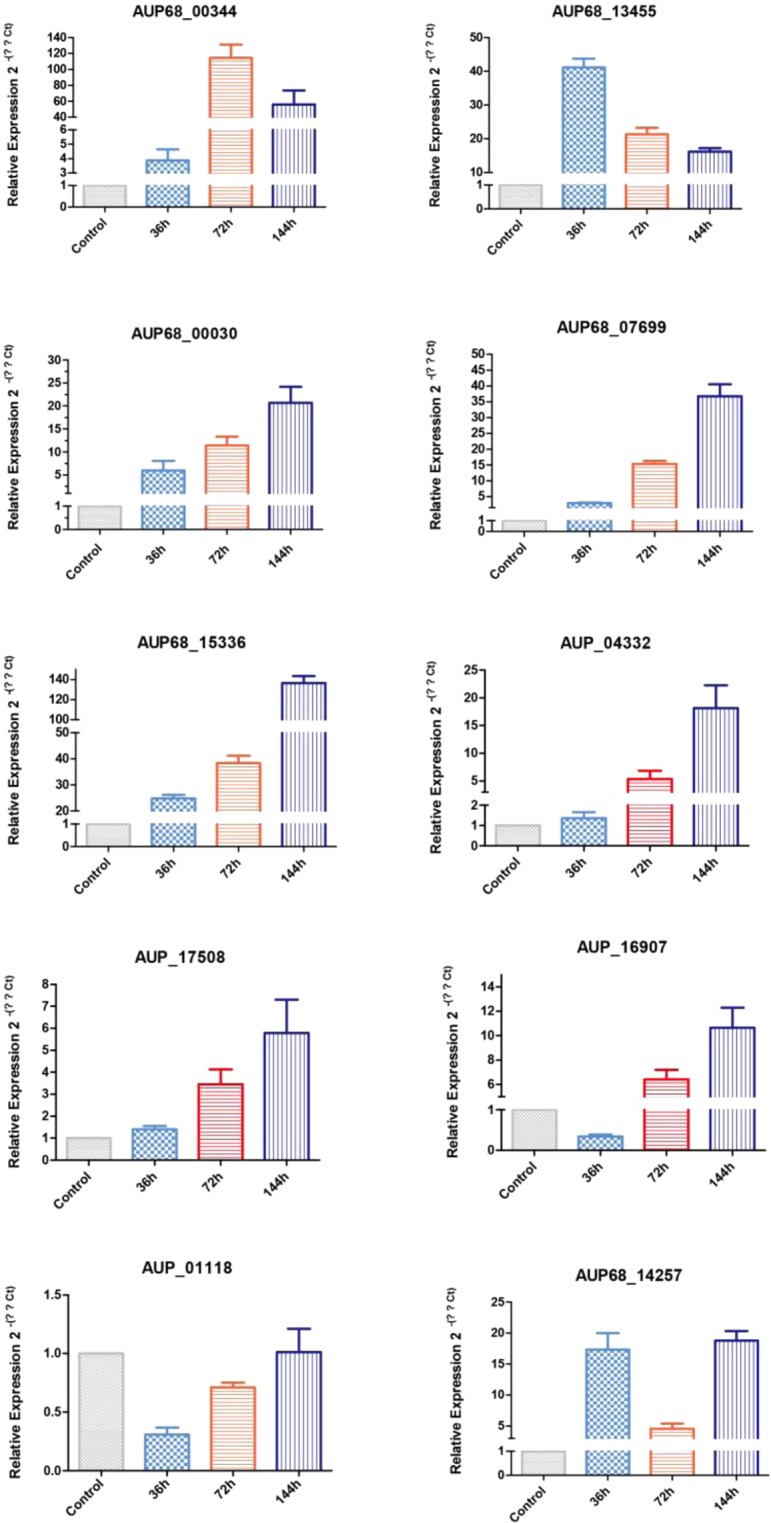
Confirmation of differential expression of secretome protein genes by qRT-PCR. Gray, green, red and purple represent infected samples at 0 h (control), 36 h, 72 h, 144 h; the control was normalized to “1”, and the ITS gene was used as a reference gene.

The expression levels of ten selected genes were significantly different at the different infection stages, with all being up-regulated during infection. *AUP68_00344* was not detected at the 36 h after infection of the transcriptome but was expressed at a low level according to qRT-PCR, and the results were consistent with the transcriptome data at the 72 h and 144 h after infection. Expression of *AUP68_13455* was highest at the 36 h after infection, decreasing with infection time. *AUP68_00030*, *AUP68_07699*, *AUP68_04332*, *AUP68_16907*, *AUP68_17508*, *AUP68_01118* and *AUP68_15336* were expressed at the lowest levels at the 36 h after infection but gradually increased with infection time; *AUP68_14257* levels were high at 36 h, and 144 h but reduced at 72 h. The results of relative gene expression were consistent with the trend of the same genes in the transcriptome (Fig. 8), and thus the transcriptomic data are reliable.

## Discussion

*I*. *robusta* is a soil-borne pathogenic fungus that causes the devastating rusty root rot disease of ginseng and is present in all ginseng growing areas^1^. This is the first report of the high-quality *I*. *robusta* genome sequence obtained using PacBio technology. We explored the molecular mechanisms of rusty root rot disease, which threatens ginseng production worldwide, through the first comprehensive genomic and transcriptomic analyses of *I*. *robusta* and its infection process. Our results defined three stages of *I*. *robusta* CD-56 infection after inoculation and one control stage. Our CD-56 transcriptomic analysis revealed that the number of expressed genes decreased at the initial infection stage and then increased gradually after inoculation. The CD-56 gene expression profiles exhibited a trend of initially suppressed expression, gradually returning to levels similar to those in the control (Fig. 3A). The number of significantly up- and down-regulated genes decreased gradually over time after inoculation (Fig. 4A). Moreover, global gene expression patterns in CD-56 changed from suppression to adaptation during its interaction with ginseng, consistent with its chronic pathogenic characteristics. In general, the gene expression patterns of CD-56 differed from those of members of its closest related genus, *Fusarium*, highlighting the diversity of plant pathogenic fungi^22,23,48^.

Secretome proteins are considered relevant to the progression and success of infection^35^. The proportion of secretome protein genes in *I*. *robusta* (2.86%) is much lower than that in species in *Fusarium*, such as *Fusarium fujikuroi*^49^ (8.78%) and *Fusarium graminearum*^50^ (10.51%), but it is similar to that of the lignocellulose-degrading basidiomycete fungus *Pleurotus ostreatus*^51^. There are 121 secretory protein genes with hits in the PHI database^36^. The gene number in *I*. *robusta* is nearly three times that in the sunflower rust fungus *Puccinia helianthi* Schw^52^, and *I*. *robusta* contains ‘increased virulence’ class genes, which are absent in *P*. *helianthi* Schw^52^. Based on dual RNA-Seq analysis, only 19 PHI genes were significantly regulated after inoculation. In particular, only one effector gene and one increased virulence gene were significantly regulated at 72 h and 144 h after inoculation, with both up-regulated at 144 h after inoculation. Both the high-virulence-related gene number and significantly regulated gene number are small, indicating that the degree of pathogenicity may be more reflected in the efficacy of toxicity.

CAZymes in fungal genomes are reported to have a strong relationship with the saprophytic lifestyle of fungi^46^. The number of CAZymes (129 genes) in the *I*. *robusta* genome is much lower than the number in most brown (~366 genes), rot (~480 genes) and soft-rot (~553 genes) fungi^53^. Indeed, only one GT family gene is identified in *I*. *robusta*, whereas there are many GT family genes in *Fusarium fujikuroi*^49^ and most brown, white and soft rot fungi^53^. Many cellulose-degrading enzymes are categorized within GH classes (59 genes) with abundance of the GH43 (7 genes) and GH28 (6 genes) families, which can catalyze the degradation of hemicellulose, and pectin^53^, in the *I*. *robusta* genome. Although GH class genes show enrichment of GH3, GH5 class enzymes are enriched in most white, brown and soft rot fungi^53^. GHs are also reported to be abundant in *F*. *fujikuroi*^49^, *F*. *virguliforme*^54^ and most white, brown and soft rot fungi^53^. *I*. *robusta* has one GH67 gene (*AUP68_07694*) that is absent in most plant pathogens, whereas some common hemicellulose classes of plant pathogenic fungi are absent in *I*. *robusta*, such as GH29 and GH44. Our dual RNA-Seq analysis indicated that many of these CAZyme genes are up-regulated during the host–pathogen interaction (Table 3), a situation similar to that in *F*. *fujikuroi*^49^ and the brown rot fungus *Postia placenta*^55^. The down-regulated CAZyme genes belong to the AA1, AA5 and CBM1 families (Fig. 6). Enrichment of the GH (7 genes), PL (5 genes) and AA (4 genes) classes is found for the regulated CAZyme genes, suggesting that these enzymes play important roles in plant cell wall degradation. However, the GH67 gene and the genes from abundant GH classes were not significantly regulated (Fig. 6). Moreover, an abundance of pectin-degrading enzyme families, CE8 (1 gene), GH28 (6 genes), PL1 (7 genes), PL3 (5 genes), and PL9 (1 gene) was observed, with 2 PL1 genes and 3 PL3 genes significantly up-regulated, which may help *I*. *robusta* to colonize root tissues. These results indicate that the role and mechanism of CAZyme genes in the saprophytic lifestyle is much different than that in most rot fungi. Nonetheless, it remains unclear whether any of these enzymes directly attack ginseng root cells.

The GO functional annotation analysis showed that the function of CD-56 DEGs changed from transport to cell aging at 72 h after inoculation (Fig. S5). In addition, our enriched pathway analysis of CD-56 DEGs indicated that the interaction response changed from pathogenicity- to disease-associated pathways and finally to sugar- and energy-associated metabolism (Table 2). DEGs encoding proteins that are located at the cytomembrane and respond to signals from pheromones, cell wall stress, high osmolarity, and starvation were initially suppressed and then recovered, with the suppression phenomenon shifting from the cytomembrane to intracellular locations. *CHS1* encodes chitin synthase, which is involved in biosynthesis of chitin, a major component of the cell wall^38,39^, and *CHS1* gene expression in CD-56 changed from suppression to recovery and finally activation after inoculation. This pattern of expression suggests that CD-56 cells gradually proliferate over time after inoculation. During the interaction between CD-56 and ginseng, gene expression patterns changed from a stress response to an adaptive response. CD-56 might halt infection before colonizing ginseng if the perception of invasion initiates PAMP-triggered immunity at the ginseng root cell surface^31^, which may be one of the reasons for its chronic pathogenesis.

To investigate the plant defense response, we constructed ginseng transcriptomes and analyzed changes after inoculation with the pathogen. The global gene expression patterns in ginseng root cells differed from those in CD-56 cells, with the former being highly related to physiological conditions (Fig. 3B). In ginseng, the number of up-regulated genes decreased and the number of down-regulated genes first increased and then decreased after inoculation with CD-56 (Fig. 4B). Overall, our profile analysis indicated that plant defenses were activated and that the defense response persisted in the ginseng root, but that the defenses were not sufficient to prevent the growth of CD-56.

We analyzed six types of defense-related genes in detail: genes in the plant–pathogen interaction pathway, those related to ROS, auxin, and ethylene, and those encoding MYB and WRKY transcription factors. We found that the gene expression patterns in ginseng tended to return to normal levels after inoculation (Fig. 7). Cell surface PRRs are involved in the primary response to invading pathogens, and the recognition of the pathogen triggers a relatively weak but broad-spectrum immune response^32^. The expression profiles of genes encoding PRRs suggested that ginseng root cells rapidly recognized the invading pathogen CD-56, though the response became weaker with time after infection (Fig. 2C). This may reflect the process of CD-56 colonization of ginseng roots^32^. Regardless, CD-56 does not fully suppress plant defenses, even during prolonged infection, as previously observed for other fungal pathogens^19^. For example, most WRKY transcription factor DEGs in ginseng were up-regulated during infection, similar to potato tubers infected with *P*. *infestans*^25^, though the defense mechanism of ginseng appears to have some unique traits. For instance, expression of genes associated with ROS production was inhibited in the ginseng root, yet such genes were shown to be strongly expressed during the compatible interaction between cacao and *M*. *perniciosa*^19^. The rapid production of ROS by plants is a hallmark of pathogen recognition and correlates with the hypersensitive response^25^. Our results provide new insight into the *I*. *robusta* infection process, and we identified genes related to the infection process in ginseng roots.

## Materials and Methods

### Isolation of the CD-56 strain and extraction of genomic DNA

A single-spore isolate of *I*. *robusta* CD-56 was isolated from rusty rot-infected roots of 3-year-old ginseng (*P*. *ginseng*) plants. The diseased samples were collected from Jilin Province, China. Strain CD-56 was cultured on potato dextrose agar (PDA) for 144 h on plates covered with cellophane. The fungus was transferred to liquid medium (potato dextrose broth) and cultured for an additional 36 h at 22 °C. Genomic DNA was extracted using DNeasy^®^ Plant Mini Kit (Qiagen, Hilden, CA; Cat# 69104).

### Genome sequencing and genome assembly

The CD-56 cell genome was sequenced by Pacific Biosciences II (Pacific Biosciences, San Francisco, CA, USA) and Illumina HiSeq3000 (Illumina, San Francisco, CA, USA) technology. We used Pacific Biosciences SMART analysis software 1.2 to generate long ‘filtered subreads’, which were high-quality reads. Filtered subreads were generated through the removal of adaptors, low-quality bases, and short reads. Here, we refer to subreads as ‘reads’ and do not refer to raw reads. After the reads were filtered, 12 SMRT cell sequencing generated 545,582 long reads, and the sum of bases was 6,064,719,683. The read N50 length was up to 16,517 bp. The SMRT cells yielded approximately 65.48-fold coverage. All PacBio reads were assembled into polished contigs using HGAP v3^56^, and PBJelly was used to fill gaps^57^. Assembly sequence errors were corrected using high-quality HiSeq reads. Two DNA libraries were constructed for Illumina sequencing, including a 250-bp pair-end library and a 3–5-kb mate-pair library. We generated 23,322,349 pair-end read pairs (2 × 101 bp) and 62,861,581 mate-pair reads (2 × 101 bp). High-quality sequencing reads are a prerequisite for precise correction of an assembled genome. To this end, we carried out a filtering process to filter low-quality reads (reads with excess “N” and low-quality scores), duplicated reads, adaptor reads, and contaminated reads. After the reads were filtered, the two libraries yielded approximately 50-fold coverage of high-quality reads (details shown in Table S6). Indel and base sequencing errors in the SMRT sequencing data were corrected using the SNP calling method^58^. The genome assembly sequences were aligned to UniVec (ftp://ftp.ncbi.nih.gov/pub/UniVec) to identify contaminating sequences that may be of adaptor, vector, and primer, among other origins using BLAST (filtering with the following criteria: e-value 10-5 and identity 90.0). The reserved sequences were aligned to the nt database to identify contaminated sequences that may be of bacterial origin using BLAST (filtering with following criteria: e-value 10-5, identity 90.0, query coverage 90, and no hits in eukaryotic genomes). Finally, we manually removed contaminated contigs using in-house programs. This Whole-Genome Shotgun project has been deposited in Genome Warehouse in BIG Data Center^59^, Beijing Institute of Genomics (BIG), Chinese Academy of Sciences (CAS), under accession number GWHAAAI00000000. The dataset is publicly accessible at https://bigd.big.ac.cn/gwh.

### Cell culture and transcriptome sequencing

Roots of 3-year-old ginseng (Damaya) were surface-sterilized with 75% alcohol and then rinsing with sterile distilled water. All samples were placed in a crisper (15 cm × 10 cm) and incubated at 22 °C for 24 h under a 12 h light/12 h dark photoperiod for sample culture conditions before inoculation using a published method^11^. Roots were inoculated by inserting two cut-off pipette tips (200 µl, BBI) with inoculum into the roots. Each pipette tip contained 30 µl of spore suspension (1 × 10^7^/ml) and was inserted into the root to a depth of 5 mm (Fig. S6). Ten ginseng roots were inoculated and incubated for 0, 36, 72, and 144 h. The diseased part of the same site was selected for subsequent trials with two replicates at each time point. A spore suspension was used as the fungal control (sample IDs, CD56_1 and CD56_2), and roots inoculated with sterile water for 0 h were used as the host control (sample IDs, Ginseng_1 and Ginseng_2). Phenotypes were observed at 36 h, 72 h, and 144 h after inoculation (sample IDs, 36 h_1 and 36 h_2, 72 h_1 and 72 h_2, and 144 h_1 and 144 h_2, respectively). A cork borer was used for sampling to ensure uniformity among the different time points. All samples were frozen in liquid nitrogen immediately after collection and stored at −80 °C. Total RNA was extracted using Qiagen Kit No. 74904 and purified using Qiagen Kit No. 08004 (Qiagen, Hilden, Germany).

All samples were sequenced with an Illumina sequencer. The HiSeq3000 paired-end DNA library with a fragment size of 500 bp was constructed using the Kapa Stranded mRNA-Seq Kit Illumina® platform (Kapa Biosystems, Boston, MA, USA) according to the kit’s protocol. This procedure generated 2 × 101 bp paired-end reads. The dual RNA-Seq raw read data reported in this paper have been deposited in Genome Sequence Archive^60^ in BIG Data Center^59^, BIG, CAS, Chinese Academy of Sciences under accession number CRA000204; the data are publicly accessible at https://bigd.big.ac.cn/gsa.

### Genome annotation

To identify repetitive sequences in the genome, RepeatMasker was used with the Repbase library (v20.08) and the species option ‘Fungi’. Repeat sequences were masked with ‘N’, and a detailed list of the repeat types was obtained. Open reading frames (ORFs) in the CD-56 genome were predicted using Augustus^61^ and FGenenSH 3.0^62^. The RNA-Seq data were incorporated into the Augustus prediction with BLAT^63^ (including iterative mapping). The predicted ORFs were validated by BLAST^64^, which was used to search for homologous genes, and the predicted CD-56 protein sequences were aligned to the NCBI nr (v20150902) protein database and nt (v20161218) nucleotide database by BLAST^64^ (e-value <= 1e-5) (v2.2.26). The best hit in the nr protein alignment was used to infer the biological function of each predicted CD-56 protein sequence. GO annotation was performed with the software Blast2GO^65^, which assigned homologous sequences aligned by BLAST in the NCBI nr database to GO terms. The CD-56 protein sequences were also compared with the KEGG GENES database^66^ by BLAST (e-value <= 1e-5) for KEGG Orthology (KO) assignments and pathway mapping. Transfer RNA (tRNA) genes in the genomic sequence were detected by tRNAscan-SE^67^. Ribosomal RNAs were identified using RNAmmer^68^. Other ncRNAs were identified based on hits in the Rfam database^69^.

### Transcriptome reconstruction and analysis

After sequencing adapters were removed and consecutive low-quality bases (quality score < 20) were trimmed from both the 5’ and 3’ end of the reads, high-quality RNA-Seq reads from CD-56 were aligned to the CD-56 draft genome, our own assembled genome (GWHAAAI00000000), using TopHat2 (v2.0.14) with default parameters. Transcripts of each sample were assembled using Cufflinks (v2.2.1), and DEGs between samples were identified by Cuffdiff (*p*-value < 0.05), a component of the Cufflinks package.

The RNA-Seq reads from the ginseng samples were also processed by removing sequencing adapters and trimming consecutive low-quality bases and then assembled into transcripts using Trinity^70^ (v2.2.0, -min 200). A set of non-redundant unigenes was obtained by keeping the longest transcript in a cluster as representative. Gene expression was quantified using RSEM (v1.2.6)^71^. Pairwise comparisons between samples were performed using the edgeR (McCarthy *et al*., 2012) package. We used *p* < 0.001 as the threshold to screen for DEGs.

For pathway and GO enrichment analyses of DEGs, the threshold of *p* < 0.01 was selected to identify significantly enriched KEGG and GO terms using the hypergeometric test.

### Analysis of effector genes

Secretome proteins of CD-56 were identified through several prediction software programs. SignalP v4.1 server^72^ was used to predict signal peptide cleavage sites and TMHMM v2.0 (http://www.cbs.dtu.dk/services/TMHMM/) to predict transmembrane helices. TargetP v1.1 (http://www.biomedsearch.com/sci/TargetP-11-server/) was employed to remove mitochondrial proteins, and ProtComp v9.0 (http://www.softberry.com/berry.phtml?topic=protcompan&group=programs&subgroup=proloc) was employed to predict the subcellular localization of fungal proteins. Proteins containing signal peptide cleavage sites but no transmembrane helices were considered to be secreted proteins^37^, and genes homologous to the predicted secretome protein genes were searched in the nr database using blastp (e-value ≤ 1e^−5^, identity ≥40%). GO functional annotations were obtained from Blast2GO^73^. Visualization of the annotation analysis results of the full-length novel protein-coding gene set was plotted in WEGO^74^. The cysteine content of the predicted secretome proteins was determined with Perl scripts written in-house. Multiple tandem repeats were predicted by T-Reks^75^. To classify associated pathogenicity genes and effector genes, the PHI database (Version 4.2) was searched for hits for the predicted secretome protein genes.

We used the Hmmscan program in the HMMER 3.0 package (with default parameters) to search each of the fungal predicted secretome proteins with the family-specific HMM profiles of CAZymes downloaded from the dbCAN database as queries^76^. The primary results were processed by the hmmscan-parser script supplied by dbCAN.

### qRT-PCR confirmation

The relative expression levels of selected secreted proteins were confirmed using qRT-PCR. cDNA was synthesized using Transcriptor First-Strand cDNA Synthesis Kit (B532445, Sangon, Shanghai, China), and 500 ng of total RNA was reverse transcribed in a 20-µl reaction mixture following DNase I (2270a, Takara, Shiga, Japan) treatment, with three replicates for each gene. Each reaction contained 2X SG Fast qPCR Master Mix (High Rox, B639273, BBI) and 1 µl of 1:10 diluted cDNA template with gene-specific primers (Table S7), and a StepOne Plus (ABI, Foster, CA, USA) was used. Relative quantitative analysis was performed using the 2^−ΔΔCt^ method with ITS as the reference gene.

## Supplementary information

Supplementary file.
