# Author Correction: Whole-genome and time-course dual RNA-Seq analyses reveal chronic pathogenicity-related gene dynamics in the ginseng rusty root rot pathogen *Ilyonectria robusta*

**DOI:** 10.1038/s41598-020-73761-2

**Published:** 2020-10-08

**Authors:** Yiming Guan, Meili Chen, Yingying Ma, Zhenglin Du, Na Yuan, Yu Li, Jingfa Xiao, Yayu Zhang

**Affiliations:** 1 Institute of Special Wild Economic Animal and Plant Science, Chinese Academy of Agricultural Sciences, Changchun, China; 2 Engineering Research Center of Chinese Ministry of Education for Edible and Medicinal Fungi, Jilin Agricultural University, Changchun, China; 3 National Genomics Data Center, Beijing Institute of Genomics, Chinese Academy of Sciences, Beijing, China; 4 BIG Data Center, Beijing Institute of Genomics, Chinese Academy of Sciences, Beijing, China; 5 CAS Key Laboratory of Genome Sciences and Information, Beijing Institute of Genomics, Chinese Academy of Sciences, Beijing, China; 6 University of Chinese Academy of Sciences, Beijing, China

Correction to: *Scientific Reports* 10.1038/s41598-020-58342-7, published online 31 January 2020

The Acknowledgements section in this Article is incorrect.

“We thank Wang Dawei, PhD, from Novo gene Co., Ltd (Beijing, China), for advice on experimental design and data analysis. This work was supported by the China Agriculture Research System (CARS-21), National Key R&D Program of China (2018YFD0201107), National Natural Science Foundation of China (31701354, 81903755), and Science and Technology Development Project of Jilin Province (20140204056YY, 20191001021XH).”

should read:

“We thank Wang Dawei, PhD, from Novo gene Co., Ltd (Beijing, China), for advice on experimental design and data analysis. This work was supported by the China Agriculture Research System (CARS-21), Key project at central government level: The ability establishment of sustainable use for valuable Chinese medicine resources (2060302), National Natural Science Foundation of China (31701354, 81903755), and Science and Technology Development Project of Jilin Province (20140204056YY, 20191001021XH).”

