# Supplementary material for: Whole-genome and time-course dual RNA-Seq analyses reveal chronic pathogenicity-related gene dynamics in the ginseng rusty root rot pathogen Ilyonectria robusta
Source: Sci Rep. 2020 Jan 31;10:1586. doi: 10.1038/s41598-020-58342-7 (PMC6994667; doi:10.1038/s41598-020-58342-7)
Supplement: Supplementary file 1 — Supplementary file. [file 41598_2020_58342_MOESM1_ESM.docx]

**Whole-genome and time-course dual RNA-Seq analyses reveal chronic pathogenicity-related gene dynamics in the ginseng rusty root rot pathogen *Ilyonectria robusta***

**Yi Ming Guan^1, 2, #^, Mei Li Chen^3, 4, 5, #^, Ying Ying Ma^1^, Zheng Lin Du^3 4, 5^, Na Yuan^3 4, 5^, Yu Li^2^, Jing Fa Xiao^3, 4, 5, 6, *^ and Ya Yu Zhang^1,*^**

*^1^* *Institute of Special Wild Economic Animal and Plant Science, Chinese Academy of Agricultural Sciences, Changchun, China.*

*^2^* *Engineering Research Center of Chinese Ministry of Education for Edible and Medicinal Fungi, Jilin Agricultural Universit*y*, Changchun, China.*

*^3^ National Genomics Data Center, Beijing Institute of Genomics, Chinese Academy of Sciences, Beijing, China*

*^4^ BIG Data Center, Beijing Institute of Genomics, Chinese Academy of Sciences, Beijing, China*

*^5^ CAS Key Laboratory of Genome Sciences and Information, Beijing Institute of Genomics, Chinese Academy of Sciences, Beijing, China*

*^6^ University of Chinese Academy of Sciences, Beijing, China*

*^#^ These authors contributed equally to this work.*

*Corresponding author: Pro. Ya Yu Zhang

E-mail: zyy1966999@yeah.net

*Corresponding author: Pro. Jing Fa Xiao

E-mail: [xiaojingfa@big.ac.cn](mailto:xiaojingfa@big.ac.cn)

**Table S1. Summary counts of PHI hits content of CD-56 secretome proteins**

| Pathogenicity Types | Gene No. | Gene(s) |
| --- | --- | --- |
| Reduced virulence | 40 | AUP68_00030, AUP68_00038, AUP68_18065, AUP68_17846, AUP68_17552, AUP68_17173, AUP68_16785, AUP68_16382, AUP68_16191, AUP68_15786, AUP68_15736, AUP68_15202, AUP68_14226, AUP68_14225, AUP68_13993, AUP68_13849, AUP68_12692, AUP68_12436, AUP68_12375, AUP68_12255, AUP68_11940, AUP68_11499, AUP68_09565, AUP68_09141, AUP68_08647, AUP68_07699, AUP68_07215, AUP68_07137, AUP68_07107, AUP68_07094, AUP68_05703, AUP68_05289, AUP68_05096, AUP68_05078, AUP68_04074, AUP68_03701, AUP68_02790, AUP68_00855, AUP68_00661, AUP68_00356 |
| Loss of pathogenicity | 3 | AUP68_12632, AUP68_07700, AUP68_07183 |
| Effector | 7 | AUP68_13832, AUP68_12101, AUP68_00794, AUP68_00664, AUP68_00344, AUP68_00172, AUP68_00090 |
| Unaffected pathogenicity | 55 | AUP68_18036, AUP68_17602, AUP68_17266, AUP68_17170, AUP68_17152, AUP68_17100, AUP68_17070, AUP68_16592, AUP68_15262, AUP68_15259, AUP68_14724, AUP68_14587, AUP68_14552, AUP68_14245, AUP68_13593, AUP68_13455, AUP68_11057, AUP68_10876, AUP68_10594, AUP68_10083, AUP68_09520, AUP68_09478, AUP68_09358, AUP68_09063, AUP68_08648, AUP68_08560, AUP68_08345, AUP68_08074, AUP68_07598, AUP68_07477, AUP68_07027, AUP68_06667, AUP68_05516, AUP68_05477, AUP68_05401, AUP68_05395, AUP68_05381, AUP68_05298, AUP68_05191, AUP68_04859, AUP68_04815, AUP68_04471, AUP68_04324, AUP68_04317, AUP68_04150, AUP68_03776, AUP68_02945, AUP68_02883, AUP68_02591, AUP68_02138, AUP68_02051, AUP68_00529, AUP68_00395, AUP68_00373, AUP68_00318 |
| Increased virulence | 3 | AUP68_16907, AUP68_08106, AUP68_01836 |
| Mixed outcome | 13 | AUP68_16775, AUP68_15852, AUP68_15336, AUP68_14982, AUP68_14261, AUP68_14257, AUP68_13524, AUP68_11568, AUP68_09858, AUP68_07733, AUP68_05552, AUP68_04298, AUP68_01497 |

**Table S2. Summary counts of CAZymes content of CD-56 secretome proteins**

| Gene family | Subfamily | Gene No. | Gene(s) |
| --- | --- | --- | --- |
| CBM | CBM1 | 5 | AUP68_00373, AUP68_01497, AUP68_04317, AUP68_04815, AUP68_07027, |
|  | CBM9 | 1 | AUP68_16839 |
|  | CBM18 | 3 | AUP68_03776, AUP68_03776, AUP68_13593, |
|  | CBM19 | 2 | AUP68_03306, AUP68_07934, |
|  | CBM32 | 1 | AUP68_10762 |
|  | CBM35 | 1 | AUP68_16632 |
|  | CBM38 | 1 | AUP68_18071 |
|  | CBM42 | 1 | AUP68_05400 |
|  | CBM50 | 1 | AUP68_00039 |
|  | CBM63 | 2 | AUP68_02316, AUP68_07708, |
| CE | CE1 | 5 | AUP68_00395, AUP68_02155, AUP68_05477, AUP68_07598, AUP68_16462, |
|  | CE4 | 2 | AUP68_03776, AUP68_13593, |
|  | CE5 | 2 | AUP68_02976, AUP68_15259 |
|  | CE8 | 1 | AUP68_09858 |
|  | CE10 | 6 | AUP68_00318, AUP68_02138, AUP68_04471, AUP68_05191, AUP68_10876, AUP68_18036, |
|  | CE12 | 1 | AUP68_14260 |
| GH | GH1 | 1 | AUP68_05180 |
|  | GH3 | 5 | AUP68_05552, AUP68_07733, AUP68_13524, AUP68_14552, AUP68_14587, |
|  | GH5 | 2 | AUP68_04815, AUP68_17508, |
|  | GH6 | 1 | AUP68_00373 |
|  | GH10 | 3 | AUP68_02051, AUP68_05298, AUP68_14261, |
|  | GH11 | 1 | AUP68_10083 |
|  | GH12 | 2 | AUP68_00172, AUP68_00344, |
|  | GH13 | 1 | AUP68_15984 |
|  | GH16 | 4 | AUP68_08058, AUP68_09747, AUP68_11855, AUP68_15249 |
|  | GH18 | 3 | AUP68_09141, AUP68_12436, AUP68_13993, |
|  | GH23 | 1 | AUP68_00694 |
|  | GH24 | 1 | AUP68_04450 |
|  | GH28 | 6 | AUP68_07367, AUP68_09666, AUP68_15262, AUP68_15404, AUP68_15852, AUP68_17152 |
|  | GH30 | 1 | AUP68_00465 |
|  | GH31 | 1 | AUP68_07183 |
|  | GH32 | 1 | AUP68_18071 |
|  | GH39 | 1 | AUP68_07207 |
|  | GH43 | 7 | AUP68_00448, AUP68_06526, AUP68_07749, AUP68_09429, AUP68_09479, AUP68_16632, AUP68_17845 |
|  | GH45 | 1 | AUP68_06267 |
|  | GH51 | 1 | AUP68_16025 |
|  | GH53 | 1 | AUP68_09598 |
|  | GH54 | 1 | AUP68_05400 |
|  | GH55 | 1 | AUP68_13510 |
|  | GH62 | 1 | AUP68_18028 |
|  | GH64 | 2 | AUP68_07960, AUP68_11390 |
|  | GH67 | 1 | AUP68_07694 |
|  | GH76 | 1 | AUP68_02927 |
|  | GH93 | 3 | AUP68_07849, AUP68_09272, AUP68_17111 |
|  | GH105 | 1 | AUP68_02802 |
|  | GH127 | 1 | AUP68_05089 |
|  | GH128 | 1 | AUP68_03376 |
|  | GH131 | 1 | AUP68_00713 |
| GT | GT69 | 1 | AUP68_02801 |
| PL | PL1 | 7 | AUP68_00855, AUP68_04298, AUP68_05078, AUP68_07699, AUP68_11568, AUP68_14225, AUP68_16775 |
|  | PL3 | 5 | AUP68_00030, AUP68_01497, AUP68_14257, AUP68_14982, AUP68_15336 |
|  | PL4 | 2 | AUP68_14066, AUP68_18041 |
|  | PL9 | 1 | AUP68_09975 |
| AA | AA1 | 1 | AUP68_08648 |
|  | AA2 | 1 | AUP68_17552 |
|  | AA3 | 1 | AUP68_13849 |
|  | AA5 | 3 | AUP68_07700, AUP68_08647, AUP68_10762, |
|  | AA7 | 2 | AUP68_05516, AUP68_09520, |
|  | AA8 | 2 | AUP68_07107, AUP68_17173, |
|  | AA9 | 9 | AUP68_00090, AUP68_02591, AUP68_03306, AUP68_07027, AUP68_08074, AUP68_08345, AUP68_14724, AUP68_14754, AUP68_17602, |

**Table S3. Overview of ginseng transcripts assembly result**

|  | Transcript | Unigene |
| --- | --- | --- |
| Total number | 224,617 | 104,708 |
| Total length (bp) | 174,992,974 | 71,407,790 |
| Mean length (bp) | 779 | 681 |
| N50 (bp) | 1,296 | 1,107 |

**Table S4. Dual RNA-Seq reads mapping rate of CD-56 & ginseng**

|  | CD-56 | | Ginseng | | Total |
| --- | --- | --- | --- | --- | --- |
| SampleID | Mappable Reads count | Mapping rate (%) | Mappable Reads count | Mapping rate (%) | Total mapping rate (%) |
| 36h_1 | 1,297,138 | 2.4% | 34,945,644 | 64.0% | 66.4% |
| 36h_2 | 151,993 | 0.3% | 30,597,246 | 64.6% | 64.9% |
| 72h_1 | 339,109 | 0.8% | 28,183,411 | 65.2% | 66.0% |
| 72h_2 | 661,350 | 2.0% | 21,767,024 | 64.2% | 66.1% |
| 144h_1 | 8,927,248 | 10.3% | 56,935,086 | 65.6% | 75.8% |
| 144h_2 | 18,103,313 | 24.3% | 47,243,380 | 63.4% | 87.8% |

**Table S5. Dual RNA-seq captures the expressed transcript repertoire of CD-56 & ginseng.**

| Type/gene symbol^a^ |  | Expression value (FPKM)^b^ | | | | log2Fold change (P_value)^#^ | | |
| --- | --- | --- | --- | --- | --- | --- | --- | --- |
|  | **Transcript** | **Control** | **36 h** | **72 h** | **144 h** | **36 h** | **72 h** | **144 h** |
| Effector (PHI) | AUP68_00344 | 0 | 0 | 67.91 | 47.12 | 1 (1) | +∞ (1.75E-03*) | +∞(1.75E-03*) |
|  | AUP68_13832 | 0.10 | 0 | 58.66 | 313.73 | -∞ (1) | 9.14 (0.12) | 11.56(0.12) |
|  | AUP68_00172 | 0.11 | 0 | 0 | 11.64 | -∞ (1) | -∞ (1) | 6.69(1) |
|  | AUP68_12101 | 0.77 | 0 | 0 | 0.27 | -∞ (1) | -∞ (1) | -1.50(1) |
|  | AUP68_00794 | 56.33 | 39.55 | 50.83 | 30.59 | -0.51 (0.67) | -0.15 (0.90) | -0.88(0.46) |
| Novel candidate effectors | AUP68_11521 | 0.51 | 0 | 0 | 0 | -∞ (1) | -∞ (1) | #NAME? |
|  | AUP68_16048 | 0.53 | 1.84 | 0 | 1.28 | 1.80 (1) | -∞ (1) | 1.28(1) |
|  | AUP68_07712 | 0.74 | 0 | 0 | 7.67 | -∞ (1) | -∞ (1) | 3.38(1) |
|  | AUP68_13033 | 1.98 | 0 | 0 | 0.68 | -∞ (1) | -∞ (1) | -1.55(1) |
|  | AUP68_05479 | 2.40 | 0 | 0 | 0.25 | -∞ (1) | -∞ (1) | -3.27(1) |
|  | AUP68_01779 | 3.02 | 0 | 0 | 4.36 | -∞ (1) | -∞ (1) | 0.53(1) |
|  | AUP68_00306 | 3.68 | 0 | 0 | 0.24 | -∞ (1) | -∞ (1) | -3.97(1) |
|  | AUP68_13384 | 57.04 | 4.15 | 0 | 3.12 | -3.78 (0.25) | -∞ (5.00E-05*) | -4.19(0.25) |
| *Cf5* | TRINITY_DN65439_c0_g1 | 0.68 | 12.90 | 36.41 | 8.21 | 4.31 (1.27E-12*) | 5.77 (3.05E-27*) | 3.59(5.49E-09*) |
|  | TRINITY_DN61492_c0_g1 | 2.31 | 0.91 | 0.04 | 0.52 | -1.20 (4.94E-03*) | -5.71 (2.10E-11*) | -2.22(1.07E-06*) |
|  | TRINITY_DN70801_c0_g2 | 2.23 | 0.15 | 0.47 | 1.91 | -2.74 (1.10E-08*) | -1.18 (0.02) | 0.91(0.02) |
| *Cf2* | TRINITY_DN49306_c0_g1 | 3 | 4.21 | 0 | 1.11 | 2.10 (1.27E-03*) | -5.80 (0.01*) | 0.15(0.90) |
|  | TRINITY_DN61648_c0_g1 | 10.11 | 26.76 | 9.18 | 5.19 | 1.47 (1.31E-02) | -0.12 (0.83) | -0.89(1.05E-02) |
|  | TRINITY_DN65460_c0_g1 | 13.78 | 5.6 | 6.68 | 25.74 | -1.24 (2.97E-03*) | -1.04 (3.59E-03*) | 0.92(3.28E-03*) |
|  | TRINITY_DN68679_c2_g1 | 5.54 | 1.83 | 3.90 | 1.35 | -1.13 (2.26E-03*) | -0.98 (1.09E-02) | -1.79(3.03E-06*) |
|  | TRINITY_DN74227_c0_g1 | 0.34 | 1.30 | 2.23 | 10.12 | 1.95 (0.02) | 2.72 (1.07E-03*) | 4.91(3.51E-13*) |
|  | TRINITY_DN66384_c1_g1 | 5.46 | 5.31 | 3.07 | 17.20 | 0.10 (0.82) | -0.71 (0.11) | 1.74(3.84E-07*) |
|  | TRINITY_DN58395_c0_g1 | 1.44 | 4.51 | 1.19 | 10.72 | 1.71 (8.14E-04*) | -0.20 (0.82) | 2.92(1.43E-10*) |
|  | TRINITY_DN69630_c1_g1 | 1.11 | 0.54 | 1.20 | 4.22 | -1.05 (0.08) | 0.13 (0.82) | 1.93(1.97E-05*) |
|  | TRINITY_DN19424_c1_g1 | 0.58 | 2.44 | 2.54 | 4.20 | 2.13 (2.71E-03*) | 2.15 (6.63E-03*) | 2.85(6.61E-05*) |
|  | TRINITY_DN60788_c0_g2 | 4.01 | 8.89 | 0.67 | 3.00 | 1.22 (1.01E-03*) | -2.57 (5.40E-06*) | -0.49(0.21) |
|  | TRINITY_DN64079_c5_g5 | 0.95 | 0.27 | 0.07 | 0.15 | -1.72 (3.78E-03*) | -3.48 (2.70E-06*) | -2.54(5.35E-06*) |
|  | TRINITY_DN62288_c0_g2 | 0.80 | 0.66 | 0.075 | 0.42 | -0.23 (0.61) | -3.21 (2.08E-07*) | -0.90(0.02) |
|  | TRINITY_DN84712_c0_g1 | 27.99 | 10.21 | 9.128 | 15.70 | -1.41 (1.52E-04*) | -1.61 (3.34E-06*) | -0.81(1.01E-02) |
|  | TRINITY_DN66652_c4_g4 | 5.92 | 3.12 | 1.91 | 3.99 | -0.88 (1.19 E-02) | -1.61 (1.81E-05*) | -0.55(0.11) |
|  | TRINITY_DN68970_c2_g1 | 8.47 | 13.38 | 20.99 | 16.77 | 0.22 (0.51) | 1.42 (1.50E-05*) | 1.09(3.72 E-04*) |
|  | TRINITY_DN66436_c0_g1 | 1.91 | 2.60 | 12.96 | 7.90 | 0.51 (0.41) | 2.78 (4.71E-08*) | 2.02(4.90E-05*) |
|  | TRINITY_DN44761_c0_g1 | 0.81 | 2.45 | 4.09 | 3.43 | 1.66 (3.14E-03*) | 2.36 (6.97E-06*) | 2.09(1.30E-05*) |
|  | TRINITY_DN68783_c2_g1 | 0.93 | 2.60 | 10.38 | 6.25 | 1.53 (0.01*) | 3.49 (5.12E-12*) | 2.75(1.47E-09*) |
|  | TRINITY_DN90951_c0_g1 | 0.49 | 0.51 | 3.40 | 5.23 | 0.11 (1) | 2.77 (3.25E-05*) | 3.39(4.59E-07*) |
|  | TRINITY_DN62772_c2_g1 | 2.08 | 3.73 | 6.54 | 17.63 | 0.90 (1.13E-02) | 1.68 (6.14E-05*) | 3.10(1.81E-19*) |
|  | TRINITY_DN58718_c0_g1 | 1.06 | 0.03 | 0.04 | 0.06 | -4.89 (2.01E-07*) | -4.52 (1.39E-06*) | -3.94(2.33E-07*) |
|  | TRINITY_DN13935_c0_g1 | 0.44 | 0.05 | 0.08 | 0.03 | -3.05 (3.30E-05*) | -2.36 (1.08E-03*) | -3.57(4.15E-07*) |
|  | TRINITY_DN72157_c5_g1 | 5.39 | 1.07 | 0.61 | 3.41 | -2.54 (5.38E-11*) | -2.59 (8.39E-12*) | -0.99(0.03) |
|  | TRINITY_DN70889_c0_g1 | 6.02 | 1.90 | 4.64 | 6.52 | -1.59 (2.78E-05*) | -0.31 (0.38) | 0.14(0.67) |
|  | TRINITY_DN67624_c2_g2 | 27.14 | 5.34 | 1.32 | 12.82 | -2.30 (1.42E-06*) | -4.31 (3.69E-18*) | -1.06(2.47 E-03*) |
|  | TRINITY_DN68261_c0_g1 | 1.71 | 0.12 | 0.40 | 0.80 | -3.71 (3.30E-17*) | -2.13 (2.78E-07*) | -1.09(4.03E-03*) |
|  | TRINITY_DN61994_c0_g1 | 1.27 | 0.08 | 0.48 | 0.42 | -3.96 (1.95E-09*) | -1.36 (6.48E-03*) | -1.60(1.89E-03*) |
|  | TRINITY_DN71472_c1_g2 | 4.09 | 0.65 | 1.15 | 1.86 | -2.25 (3.75E-09*) | -1.58 (3.04E-05*) | -0.95(7.45E-03*) |
|  | TRINITY_DN66229_c8_g2 | 0.56 | 0.05 | 0.10 | 0.23 | -3.61 (2.26E-06*) | -2.51 (3.32E-04*) | -1.26(1.19E-02) |
|  | TRINITY_DN64599_c7_g6 | 11.55 | 3.45 | 3.72 | 4.00 | -1.68 (2.92E-05*) | -1.60 (7.68E-05*) | -1.17(9.54E-04*) |
|  | TRINITY_DN65968_c0_g2 | 6.37 | 0.07 | 0.15 | 0.56 | -6.07 (3.53E-24*) | -5.68 (1.08E-20*) | -3.42(6.25E-12*) |
|  | TRINITY_DN65420_c0_g1 | 5.95 | 1.18 | 1.13 | 3.08 | -2.10 (8.17E-08*) | -2.58 (6.76E-11*) | -0.88(7.72 E-03*) |
|  | TRINITY_DN59282_c2_g1 | 1.36 | 0.21 | 0.03 | 0.18 | -2.63 (2.72E-06*) | -5.40 (3.15E-10*) | -2.88(2.44E-07*) |
|  | TRINITY_DN67678_c1_g1 | 12.93 | 81.13 | 34.12 | 30.88 | 3.33 (6.19E-21*) | 2.04 (5.21E-08*) | 1.89(3.01E-09*) |
|  | TRINITY_DN55760_c0_g1 | 11.23 | 87.63 | 79.60 | 49.08 | 3.05 (1.68E-10*) | 2.87 (9.52E-09*) | 2.13(3.05E-06*) |
|  | TRINITY_DN71904_c3_g1 | 4.94 | 44.98 | 17.64 | 31.68 | 3.23 (1.68E-21*) | 1.86 (6.13E-06*) | 2.71(4.37E-18*) |
|  | TRINITY_DN67471_c0_g2 | 4.01 | 62.24 | 18.82 | 20.54 | 4.03 (2.97E-15*) | 2.27 (2.76E-06*) | 2.35(5.72E-08*) |
|  | TRINITY_DN86782_c0_g1 | 1.05 | 34.64 | 29.97 | 17.03 | 5.09 (3.86E-23*) | 4.87 (5.37E-14*) | 3.96(7.92E-12*) |
|  | TRINITY_DN72289_c2_g3 | 27.40 | 93.31 | 47.25 | 64.87 | 1.82 (9.82E-08*) | 0.86 (0.03) | 1.37(4.61E-06*) |
|  | TRINITY_DN69902_c0_g1 | 0.49 | 9.20 | 15.89 | 12.01 | 4.28 (2.55E-16*) | 5.02 (9.55E-24*) | 4.61(3.17E-22*) |
|  | TRINITY_DN35520_c0_g2 | 0.40 | 17.61 | 1.03 | 0.57 | 5.23 (1.21E-06*) | 1.19 (1) | 0.45(1) |
|  | TRINITY_DN62965_c0_g1 | 3.38 | 30.25 | 4.03 | 3.42 | 3.21 (8.90E-21*) | 0.25 (0.61) | 0.01(0.99) |
|  | TRINITY_DN69995_c2_g1 | 0.55 | 6.11 | 8.16 | 4.46 | 3.51 (7.31E-10*) | 3.88 (2.22E-14*) | 2.98(4.12E-07*) |
|  | TRINITY_DN72093_c0_g2 | 28.34 | 130.01 | 121.78 | 87.69 | 1.84 (4.79E-06*) | 1.77 (5.89E-06*) | 1.48(3.90E-05*) |
|  | TRINITY_DN51974_c3_g1 | 0.57 | 2.40 | 0.40 | 0.78 | 2.13 (3.22E-05*) | -0.41 (0.55) | 0.49(0.35) |
|  | TRINITY_DN67471_c2_g1 | 1.15 | 11.90 | 10.15 | 13.79 | 3.43 (1.78E-15*) | 3.17 (4.03E-08*) | 3.59(4.94E-17*) |
|  | TRINITY_DN69292_c0_g1 | 2.00 | 9.13 | 3.02 | 3.80 | 2.24 (1.50E-10*) | 0.62 (0.08) | 0.96(4.77 E-03*) |
|  | TRINITY_DN60007_c0_g1 | 37.27 | 101.15 | 75.05 | 87.60 | 1.49 (6.33E-06*) | 1.03 (2.06E-03*) | 1.26(1.06E-04*) |
|  | TRINITY_DN67915_c4_g2 | 14.24 | 48.55 | 60.41 | 66.97 | 1.93 (2.27E-06*) | 2.31 (9.30E-09*) | 2.51(1.66E-10*) |
|  | TRINITY_DN29244_c0_g1 | 0.44 | 3.34 | 2.75 | 3.54 | 2.99 (8.38E-05*) | 2.67 (3.18 E-03*) | 3.01(1.08E-05*) |
|  | TRINITY_DN64574_c0_g1 | 2.95 | 51.85 | 44.85 | 28.17 | 4.28 (3.37E-24*) | 4.06 (1.16E-20*) | 3.46(3.33E-18*) |
|  | TRINITY_DN64981_c0_g1 | 1.73 | 9.88 | 10.69 | 12.29 | 2.56 (2.07E-12*) | 2.64 (4.22E-14*) | 2.84(2.69E-16*) |
|  | TRINITY_DN69995_c0_g1 | 0.52 | 7.77 | 11.26 | 4.97 | 3.93 (3.93E-13*) | 4.42 (1.55E-21*) | 3.23(2.19E-12*) |
|  | TRINITY_DN69236_c5_g1 | 19.75 | 78.36 | 22.55 | 76.09 | 2.05 (1.15E-09*) | 0.27 (0.44) | 1.90(3.12E-09*) |
|  | TRINITY_DN51171_c0_g1 | 0.34 | 2.57 | 6.84 | 4.32 | 2.96 (2.79E-08*) | 4.33 (2.78E-20*) | 3.65(2.04E-12*) |
|  | TRINITY_DN66069_c3_g1 | 0.50 | 28.32 | 20.74 | 10.70 | 5.86 (7.18E-36*) | 5.37 (3.77E-26*) | 4.40(2.11E-21*) |
|  | TRINITY_DN44905_c0_g1 | 0.63 | 5.38 | 1.50 | 4.22 | 3.13 (6.67E-11*) | 1.30 (0.06) | 2.74(4.39E-09*) |
|  | TRINITY_DN51974_c2_g1 | 0.58 | 4.61 | 0.92 | 1.82 | 3.04 (6.40E-07*) | 0.66 (0.42) | 1.67(1.25 E-02) |
| *Cf4* | TRINITY_DN63742_c0_g2 | 1.21 | 4.63 | 3.71 | 3.35 | 1.99 (2.35E-05*) | 1.68 (1.49E-03*) | 1.46(2.27E-03*) |
| *CERK1* | TRINITY_DN67119_c5_g1 | 13.65 | 82.62 | 49.49 | 34.02 | 2.05 (2.72E-10*) | 2.35 (1.92E-11*) | 1.78(1.05E-08*) |
| *EIX1/2* | TRINITY_DN63105_c0_g1 | 8.4 | 2.75 | 0.94 | 2.04 | -1.44 (0.02) | -3.14 (4.04E-09*) | -2.24(7.65E-08*) |
|  | TRINITY_DN64602_c0_g1 | 9.30 | 16.22 | 27.41 | 28.56 | 0.85 (0.01*) | 1.58 (1.77E-06*) | 1.64(2.57E-07*) |
|  | TRINITY_DN58140_c0_g1 | 1.31 | 0 | 0.14 | 0.87 | -7.33 (8.27E-07*) | -3.19 (4.74E-04*) | -0.61(0.34) |
|  | TRINITY_DN71324_c1_g2 | 7.69 | 2.48 | 6.16 | 20.84 | -1.71 (8.47E-06*) | -0.46 (0.26) | 1.42(6.79E-05*) |
|  | TRINITY_DN61792_c1_g1 | 0.62 | 0.04 | 0.06 | 0.10 | -3.83 (2.69E-06*) | -3.42 (1.55E-04*) | -2.61(2.94E-05*) |
|  | TRINITY_DN67032_c2_g1 | 21.79 | 7.13 | 6.06 | 11.49 | -1.56 (1.24E-06*) | -1.82 (4.91E-07*) | -0.91(4.50E-03*) |
|  | TRINITY_DN43743_c0_g1 | 0.46 | 32.04 | 12.40 | 7.40 | 6.19 (2.09E-18*) | 4.78 (1.63E-10*) | 4.06(2.16E-11*) |
|  | TRINITY_DN55416_c0_g1 | 9.38 | 38.91 | 47.42 | 28.96 | 2.15 (4.06E-09*) | 2.38 (8.91E-11*) | 1.62(6.36E-06*) |
|  | TRINITY_DN65502_c2_g1 | 12.44 | 325.44 | 107.19 | 90.90 | 4.75 (4.02E-28*) | 3.13 (4.25E-14*) | 2.90(3.04E-14*) |
|  | TRINITY_DN55295_c1_g1 | 0.91 | 24.27 | 3.02 | 0.85 | 4.79 (5.77E-16*) | 1.76 (0.01*) | -0.02(1) |
|  | TRINITY_DN66187_c1_g1 | 0.64 | 5.71 | 5.69 | 8.32 | 3.22 (3.46E-12*) | 3.19 (3.62E-08*) | 3.73(1.98E-21*) |
| *XA21* | TRINITY_DN39576_c1_g1 | 0.61 | 3.97 | 0 | 0.26 | 2.76 (5.11E-05*) | -4.85 (0.08) | -1.13(0.51) |
|  | TRINITY_DN63742_c1_g1 | 0.85 | 4.34 | 3.59 | 3.31 | 2.39 (2.09E-08*) | 2.08 (1.78E-06*) | 1.96(6.33E-07*) |
|  | TRINITY_DN60000_c1_g1 | 0.97 | 9.53 | 5.53 | 6.41 | 3.36 (5.41E-12*) | 2.53 (4.09E-06*) | 2.72(5.39E-09*) |
|  | TRINITY_DN60752_c1_g1 | 3.11 | 17.82 | 19.60 | 9.40 | 2.84 (2.51E-12*) | 2.47 (2.30E-09*) | 1.85(1.83E-06*) |
| *CEBiP* | TRINITY_DN10107_c0_g1 | 19.94 | 134.09 | 104.90 | 83.50 | 2.79 (4.56E-08*) | 2.40 (1.60E-06*) | 2.10(1.04E-05*) |

^a^ The transcripts that are assigned to a same gene symbol are based on which transcripts hit to a same ko No. in KEGG database. Gene expression level is defined as the sum of all transcript expression values.

^b^ A transcript expression level (FPKM) is defined as the average of the transcript expression values of the two replicate samples.

^#^ Log2 fold changes are calculated as Cuffdiff done over 2 biological replicates, comparing to control condition. The values in parentheses represent the p values.

^*^ The asterisk means the gene/transcript expression value under an infection time was significantly different from the control time.

**Table S6. Genome sequencing details of CD-56.**

1. **PacBio sequencing.**

|  | Cells | Polymerase Reads | Polymerase Read Bases (bp) | Polymerase Read N50 (bp) | Polymerase Read Length (bp) |
| --- | --- | --- | --- | --- | --- |
| CD-56 | 12 | 545,582 | 6,064,719,683 | 16,517 | 11,116 |

1. **High quality reads sequencing preparation.**

| Insert size | Raw read pairs | High quality read pairs | High quality read pairs ratio | Estimated genome coverage^1^ |
| --- | --- | --- | --- | --- |
| 250 bp | 23,322,349 | 20,512,150 | 87.95% | 70 X |
| 2,500 bp | 62,861,581 | 42,016,130 | 66.84% | 144 X |

1. **Estimated genome coverage is calculated as base pairs of high quality reads/real genome assembly size.**

**Table S7. Secretome protein genes and Primers for qPCR**

| **Gene type** | **Gene ID** | **Primer sequences** | **Base size** |
| --- | --- | --- | --- |
| Secretome protein | *AUP68_00344* | F: 5’-CTTCCACCACAGCCAAGAAC-3’ | 111 bp |
|  |  | R: 5’- CCAGGACAGTAGGAGTAGCAAT-3’ |  |
| Secretome protein | *AUP68_13455* | F: 5’- CGAGGTTCCTGAGGAGACTAC-3’ | 124 bp |
|  |  | R: 5’- CTTGGTGGTGATGGAGACAAC-3’ |  |
| Secretome protein | *AUP68_00030* | F: 5’- CGTCTGGTGGAAGAAGGTCT -3’ | 113 bp |
|  |  | R: 5’- CGTTGTGCTGGATGATCTTGT -3’ |  |
| Secretome protein | *AUP68_07699* | F: 5’- CGCCGTCAACAACTACTTCA -3’ | 106 bp |
|  |  | R: 5’- GATGGTGTCAACGCTCTCAA -3’ |  |
| Secretome protein | *AUP68_14257* | F: 5’- CATTGGTGGCGGTGCTTA -3’ | 96 bp |
|  |  | R: 5’- CCGTAGTCGTTGGCGTAGAA -3’ |  |
| Secretome protein | AUP68_15336 | F: 5’- CTCCGACGACAACACTGATG -3’ | 108 bp |
|  |  | R: 5’- TCTTGGTCCTCCTCCTCATTATC -3’ |  |
| Secretome protein | AUP68_01118 | F: 5’- GAACCATCAAGACTGTGACGATT -3’ | 135 bp |
|  |  | R: 5’- CTATGACGCCAACCACGATAC -3’ |  |
| Secretome protein | AUP68_16907 | F: 5’- GTCCAACATCCTCTCTATCTTCAC -3’ | 142 bp |
|  |  | R: 5’- GGTCATCGTAGCCAGTATCATAAG -3’ |  |
| Secretome protein | AUP68_17508 | F: 5’- CGAGAATGACGGCTTCAATGTT -3’ | 119bp |
|  |  | R: 5’- AGGACGCTGTTGACGATGTA -3’ |  |
| Secretome protein | AUP68_04432 | F: 5’- GCTCTGCCATCTGCTCATAC -3’ | 145 bp |
|  |  | R: 5’- CGACCTCATCCTCCTCCTTAA -3’ |  |
| Reference gene | ITS | F: 5’-GGAAGTAAAAGTCGTAACAAGG -3’ | 560 bp |
|  |  | R: 5’- TCCTCCGCTTATTGATATGC -3’ |  |

**A.**

**
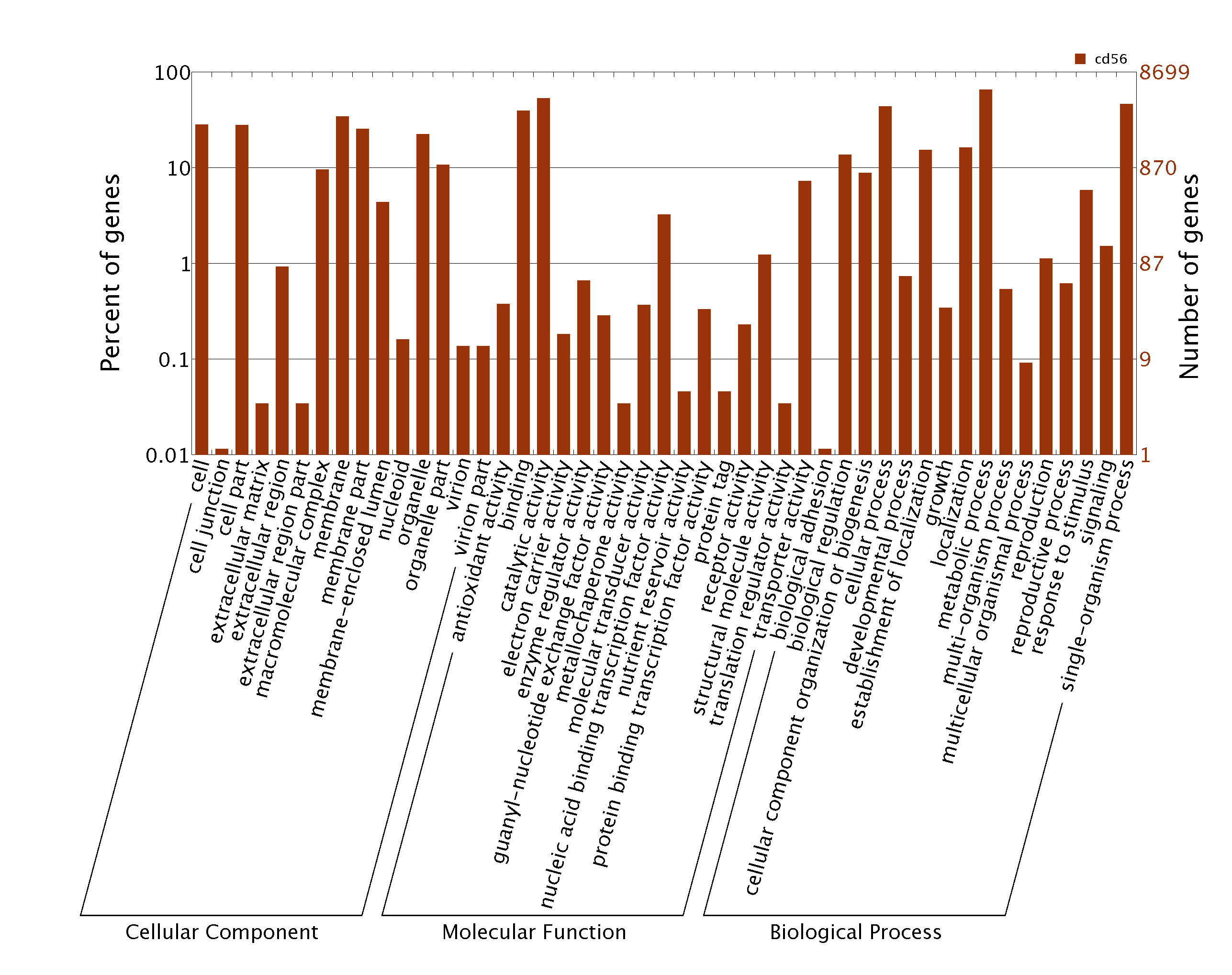
**

B.


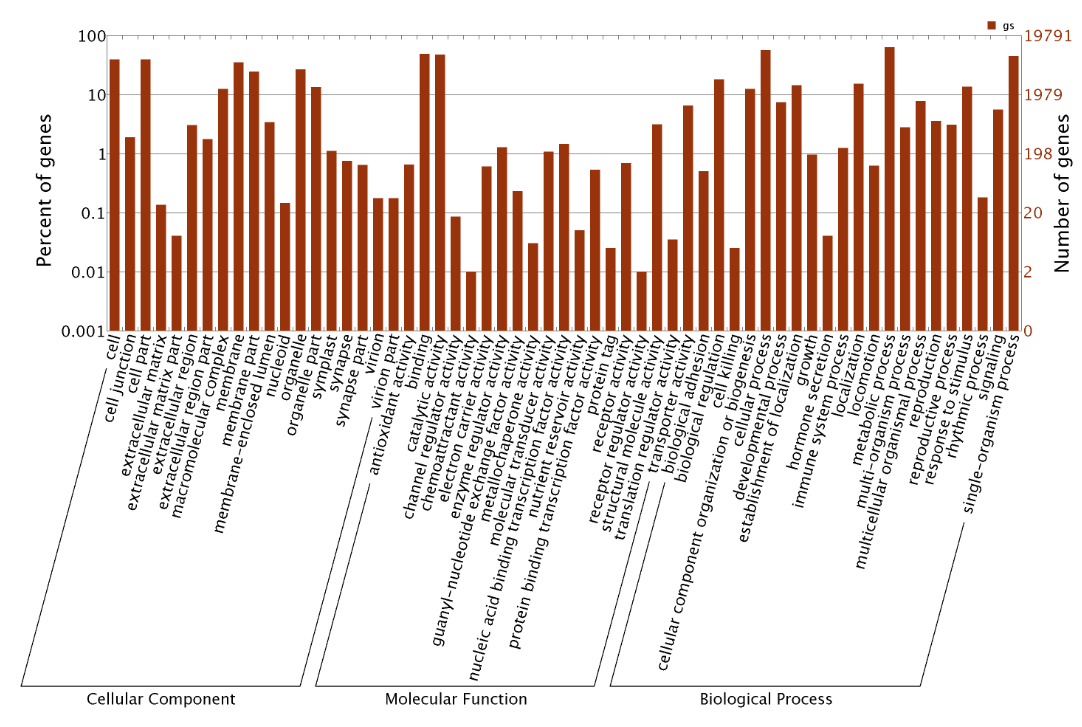


**Fig. S1. GO functional annotation of the CD-56 genome and ginseng assembled sequences.** (A) GO functional annotation of the CD-56 genome; (B) GO functional annotation of the ginseng assembled sequences.


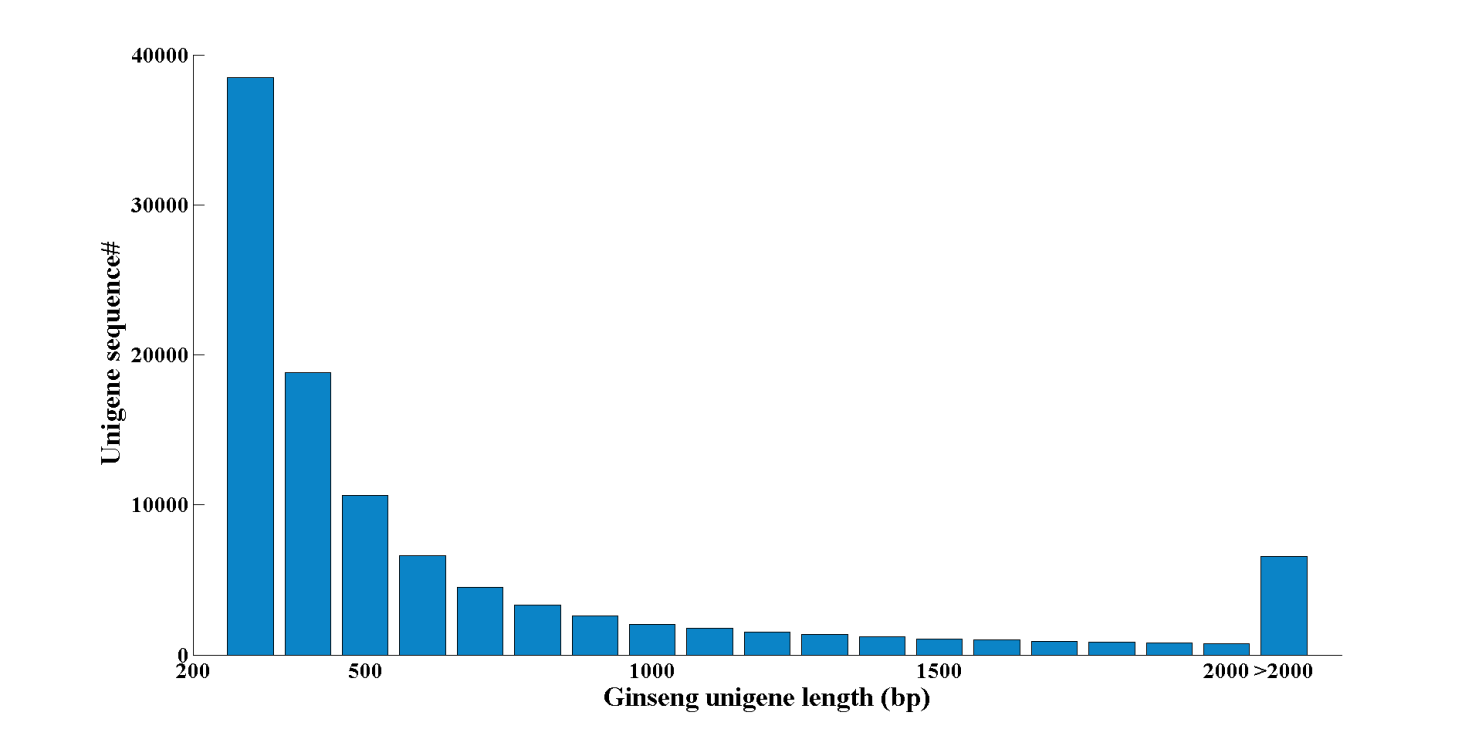


**Fig. S2. The length distribution of ginseng unigenes.** The X-axis lists the length of ginseng unigenes, and the Y-axis lists shows the number of unigenes with that size.

A.


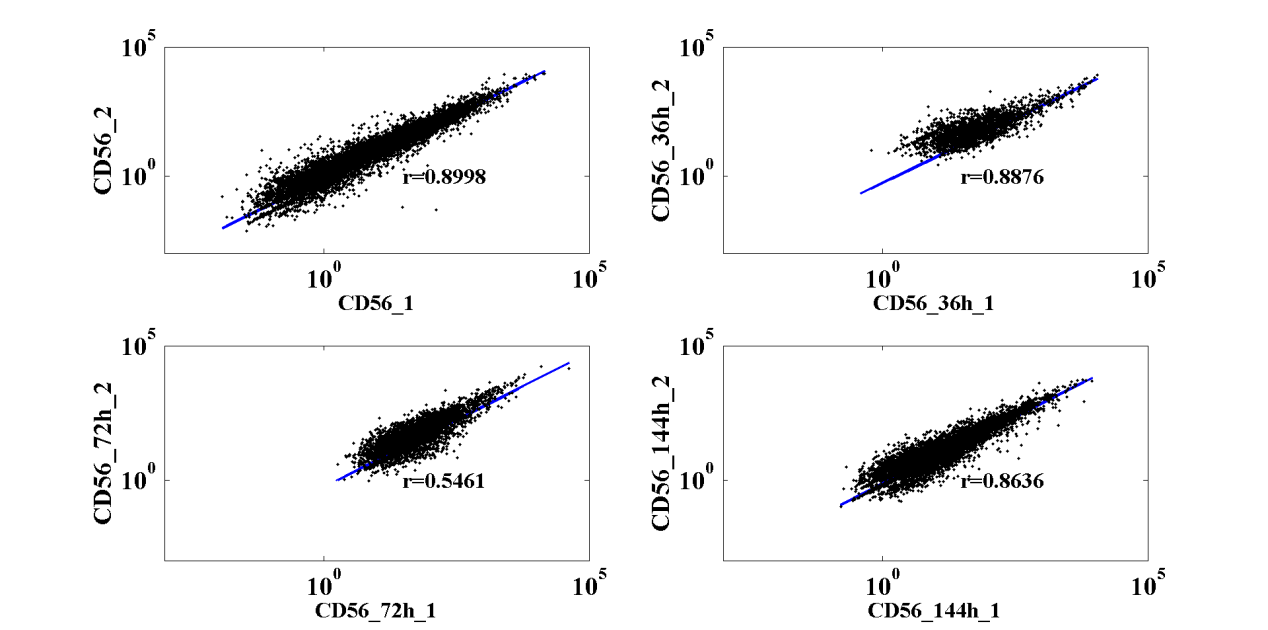


B.


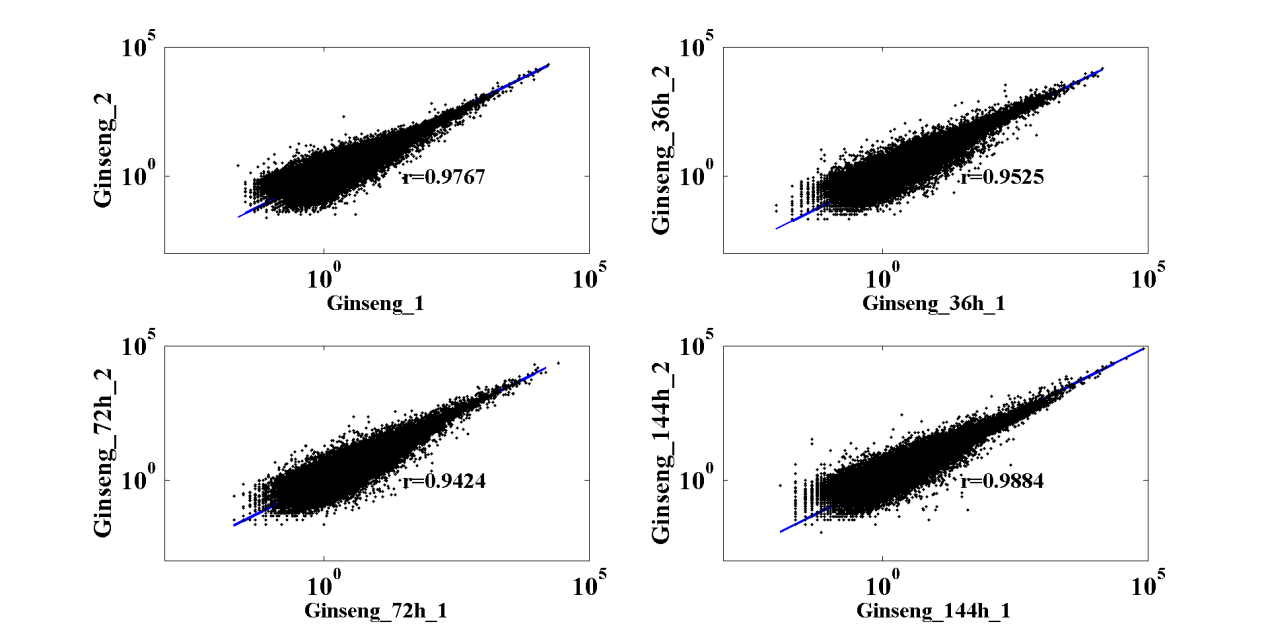


**Fig. S3. Pearson correlation coefficient scatter plots between two replicates.** A. Pearson’s correalation coefficient scatter plots for CD56; B Pearson’s correalation coefficient scatter plots for Ginseng.

A.


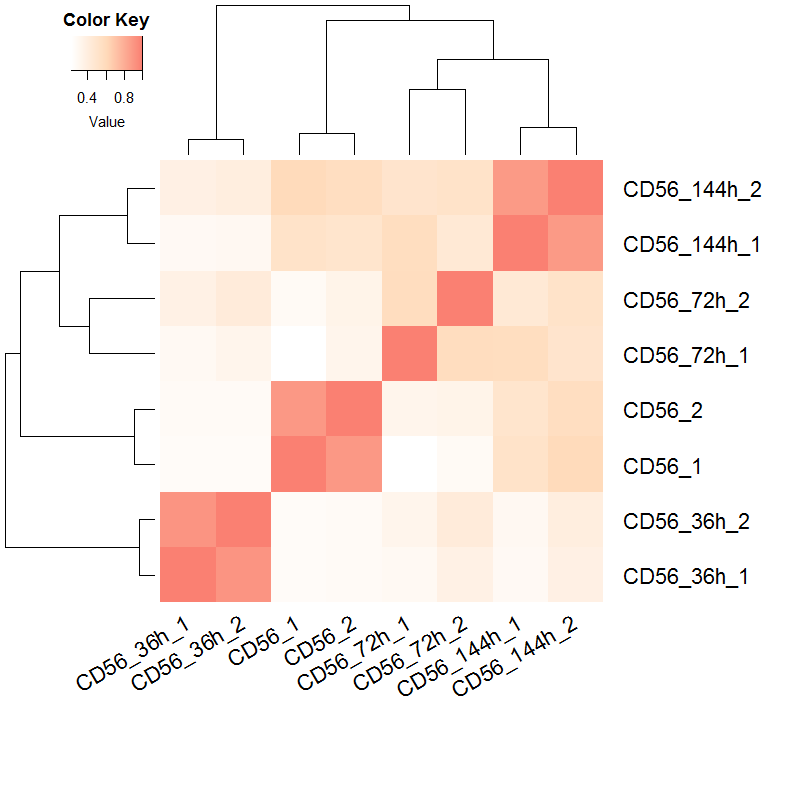


B.


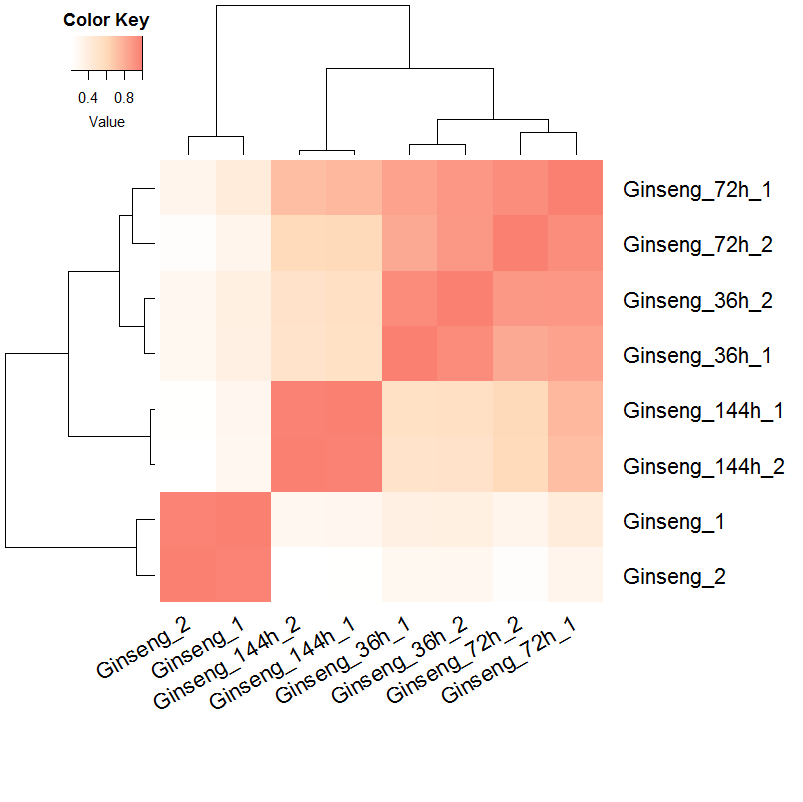


**Fig. S4. DEG expression profiles hierarchical cluster analysis based on Pearson correlation.** Pearson correlation of DEG expression profiles is used to define the DEG gene expression profiles similarity of 8 different samples. A. CD-56 DEG expression profile hierarchical cluster analysis; B. Ginseng DEG expression profile hierarchical cluster analysis.

A.


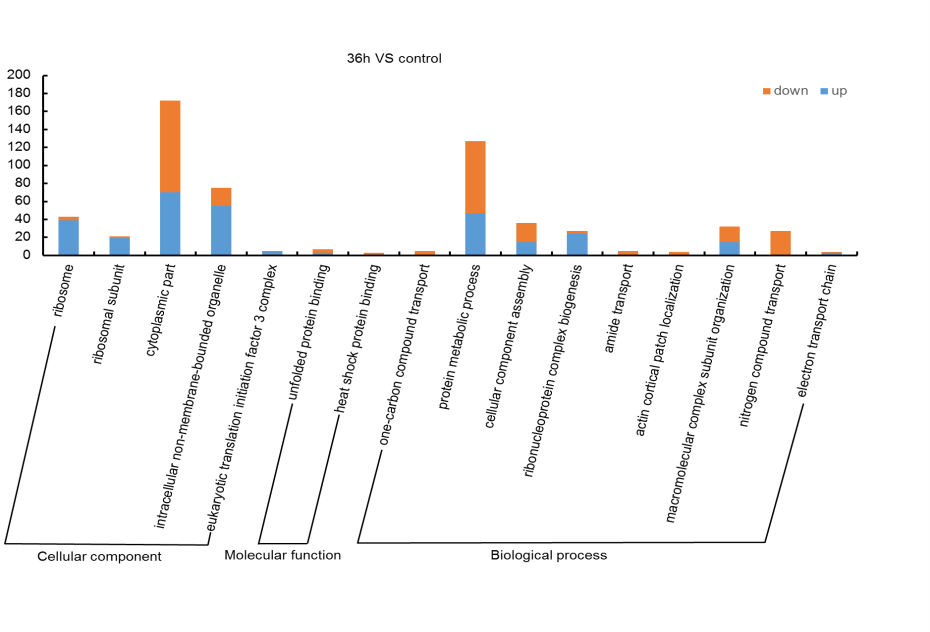


B.


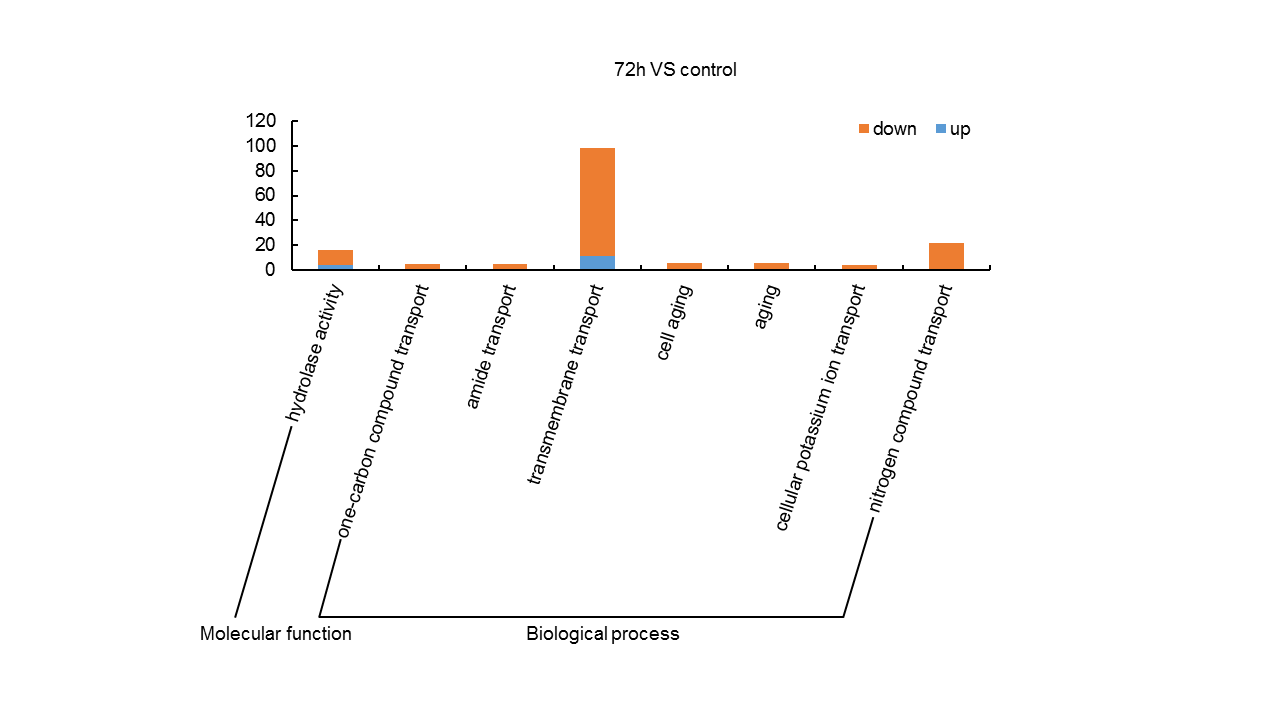


C.


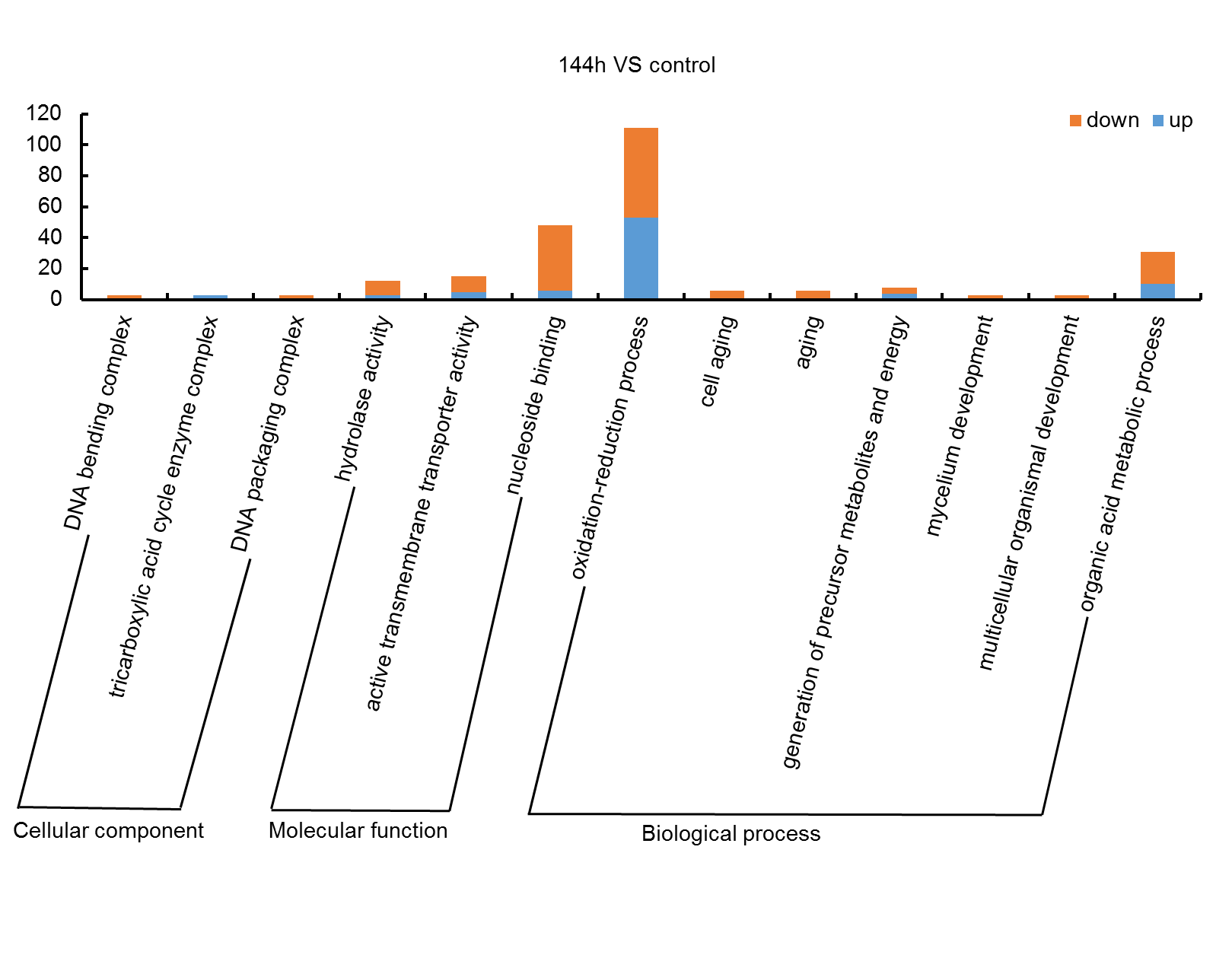


**Fig. S5. GO functional enrichment analysis of the CD-56 DEGs.** (A) GO functional enrichment analysis of the CD-56 DEGs at the 36 hours infection stage; (B) GO functional enrichment analysis of the CD-56 DEGs at the 72 hours infection stage; (C) GO functional enrichment analysis of the CD-56 DEGs at the 144 hours infection stage.


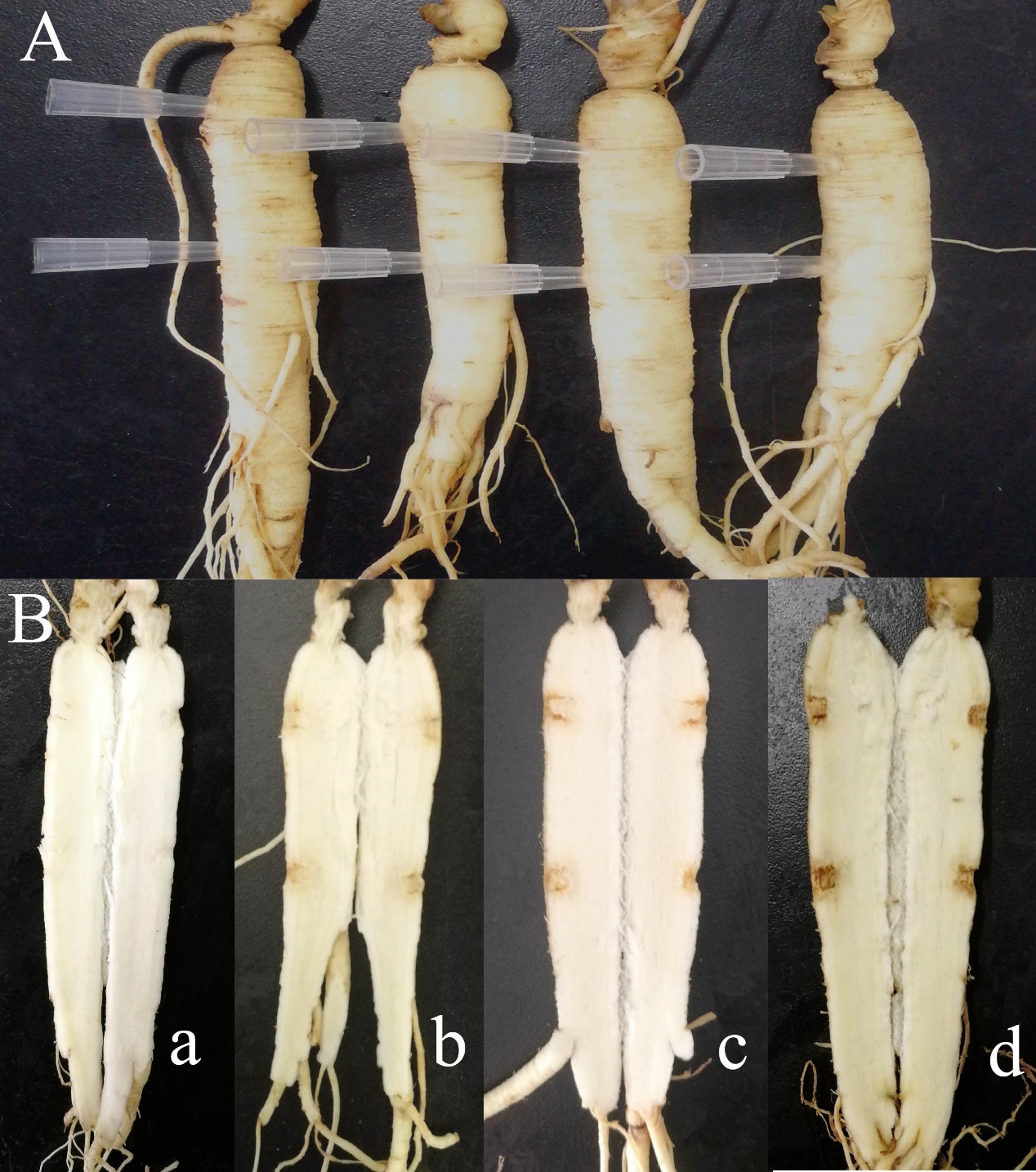


**Fig. S6. Symptoms of 3-year old ginseng roots inoculated with *Ilyonectria robusta*.** (A) 3-year old ginseng roots inoculated with *Ilyonectria robusta* for 0 hours; (B) Longitudinal section of the inoculation centerline. a. 0 h (Control); b. 36 h; c. 72 h; d. 144 h.
